# Periodontal Inflammation-Triggered by Periodontal Ligament Stem Cell Pyroptosis Exacerbates Periodontitis

**DOI:** 10.3389/fcell.2021.663037

**Published:** 2021-04-01

**Authors:** Qin Chen, Xingguang Liu, Dingyu Wang, Jisi Zheng, Lu Chen, Qianyang Xie, Xiaohan Liu, Sujuan Niu, Guanlin Qu, Jianfeng Lan, Jing Li, Chi Yang, Duohong Zou

**Affiliations:** ^1^Shanghai Ninth People’s Hospital, Shanghai Jiao Tong University School of Medicine; College of Stomatology, Shanghai Jiao Tong University; National Center for Stomatology; National Clinical Research Center for Oral Diseases; Shanghai Key Laboratory of Stomatology; Research Unit of Oral and Maxillofacial Regenerative Medicine, Chinese Academy of Medical Sciences, Shanghai, China; ^2^National Key Laboratory of Medical Immunology, Navy Military Medical University, Shanghai, China; ^3^State Key Laboratory of Pharmaceutical Biotechnology and Ministry of Education, Key Laboratory of Model Animal for Disease Study, Model Animal Research Center, Nanjing University, Nanjing, China; ^4^College of Stomatology, Inner Mongolia Medical University, Hohhot, China; ^5^Liaoning Provincial Key Laboratory of Oral Diseases, Department of Oral and Maxillofacial Surgery, School and Hospital of Stomatology, China Medical University, Shenyang, China; ^6^Guangxi Key Laboratory of Molecular Medicine in Liver Injury and Repair, The Affiliated Hospital of Guilin Medical University, Guilin, China; ^7^Shandong Provincial Key Laboratory of Oral Tissue Regeneration, School of Stomatology, Shandong University, Jinan, China

**Keywords:** GSDMD, IL-1β, PDLSC, periodontitis, pyroptosis

## Abstract

Periodontitis is an immune inflammatory disease that leads to progressive destruction of bone and connective tissue, accompanied by the dysfunction and even loss of periodontal ligament stem cells (PDLSCs). Pyroptosis mediated by gasdermin-D (GSDMD) participates in the pathogenesis of inflammatory diseases. However, whether pyroptosis mediates PDLSC loss, and inflammation triggered by pyroptosis is involved in the pathological progression of periodontitis remain unclear. Here, we found that PDLSCs suffered GSDMD-dependent pyroptosis to release interleukin-1β (IL-1β) during human periodontitis. Importantly, the increased IL-1β level in gingival crevicular fluid was significantly correlated with periodontitis severity. The caspase-4/GSDMD-mediated pyroptosis caused by periodontal bacteria and cytoplasmic lipopolysaccharide (LPS) dominantly contributed to PDLSC loss. By releasing IL-1β into the tissue microenvironment, pyroptotic PDLSCs inhibited osteoblastogenesis and promoted osteoclastogenesis, which exacerbated the pathological damage of periodontitis. Pharmacological inhibition of caspase-4 or IL-1β antibody blockade in a rat periodontitis model lead to the significantly reduced loss of alveolar bone and periodontal ligament damage. Furthermore, Gsdmd deficiency alleviated periodontal inflammation and bone loss in mouse experimental periodontitis. These findings indicate that GSDMD-driven PDLSC pyroptosis and loss plays a pivotal role in the pathogenesis of periodontitis by increasing IL-1β release, enhancing inflammation, and promoting osteoclastogenesis.

## Introduction

Periodontitis is one of the most prevalent infectious human inflammatory diseases and is distinguished by the progressive destruction of the tooth-supporting tissues and by the inflammatory reaction associated with gram-negative anaerobic bacteria (such as *Porphyromonas gingivalis* and *Treponema denticola*) in dental biofilms (Pihlstrom et al., 2005; Hajishengallis, 2015). Under this sustained inflammatory condition, the periodontal tissues (including alveolar bone, periodontal ligament, and root cementum) are destroyed, and the regeneration function of these attachment apparatuses is damaged, eventually leading to tooth loss and chewing dysfunction (Kinane et al., 2017). Recently, multiple studies have revealed that periodontitis accelerates the initiation or progression of various systemic diseases, including diabetes, cardiovascular disease, and Alzheimer’s disease, through periodontal pathogen-induced inflammation (Polak and Shapira, 2018; Aoyama et al., 2019; Dominy et al., 2019). To date, the underlying mechanisms of inflammation amplification and persistence in periodontitis remain to be elucidated.

Periodontal ligament stem cells (PDLSCs) are a population of mesenchymal stem cells (MSCs) located in the periodontal ligament that show high self-renewal capability and have multiple differentiation potentials to regenerate lost/damaged periodontal tissues (Seo et al., 2004). Although PDLSCs have exhibited remarkable reconstruction effects in artificially created bone defects in various animal models, the regeneration of periodontal tissues does not achieve the desired effect in periodontitis-caused tissue loss (Liu et al., 2008; Mrozik et al., 2013; Chen F. M. et al., 2016). Increasing evidence indicates that the inflammatory environment caused by periodontitis could inhibit the differentiation potential of PDLSCs, and the loose pluripotency of PDLSCs is associated with the activation of NF-κB, mitogen-activated protein kinase (MAPK), and BMP/Smad signaling pathways by inflammatory cytokines (Mao et al., 2016). In addition, the expression of the inflammatory cytokine IL-1β is significantly increased in periodontitis tissues, and an *in vitro* study demonstrated that IL-1β-treated PDLSCs showed impaired osteogenesis potential (Fawzy El-Sayed et al., 2019). However, the molecular mechanism of PDLSC dysfunction and loss in periodontitis remains unclear, and whether abnormal PDLSCs are involved in the pathogenesis of periodontitis is also unknown.

Increasing evidence indicates that pro-IL-1β is processed into its biologically active form IL-1β during pyroptosis, which is a critical mechanism for host defense against infection (He et al., 2015). Pyroptosis, a pro-inflammatory form of cell death, is characterized by the activation of inflammatory caspases, cell swelling, and the release of the GSDMD-N terminus (1–275 aa) to form pores (10–14 nm) in the plasma membrane (Liu et al., 2016). This process results in the massive release of cellular contents, including danger-associated molecular patterns (DAMPs) and cytokines (IL-1β and IL-18), which trigger a robust inflammatory response (Man et al., 2017). Based on the different stimuli and inflammatory caspases, pyroptosis is divided into canonical and non-canonical pyroptosis (Aglietti and Dueber, 2017). For canonical pyroptosis, canonical inflammasomes (including NLRP3, NLRC4, AIM2, and Pyrin) activate caspase-1 to cleave both GSDMD and pro-IL-1β under DAMP stimulation (Xia et al., 2020). In non-canonical pyroptosis, cytoplasmic LPS directly activates caspase-4 (mouse counterpart caspase-11) to cleave GSDMD, which is independent of inflammasomes and caspase-1 (Kesavardhana et al., 2020). Although most pyroptosis studies focus on the role of pyroptosis in the pathogenesis of systemic inflammatory diseases (such as sepsis and inflammatory bowel disease), some clinical studies have reported a correlation of pyroptosis with periodontitis (Yamaguchi et al., 2017; García-Hernández et al., 2019; Ma et al., 2020). Additionally, Jun et al. (2018) have shown that human gingival fibroblasts infected with the periodontal pathogen *Treponema denticola* demonstrated obvious pyroptosis, accompanied by the activation of caspase-4 and the release of IL-1β. Moreover, increased expression of NLRP3 and caspase-1 were reported in human MSCs derived from the human umbilical cord under lipopolysaccharide (LPS) treatment (Wang et al., 2017). Whether PDLSCs undergo pyroptosis during periodontal pathogen infection is not known. How PDLSC pyroptosis is involved in the pathogenesis of periodontitis is also not clear.

In this study, combining human periodontitis specimens (*n* = 87), we found that PDLSCs underwent GSDMD-mediated pyroptosis with typical cell swelling and IL-1β release during periodontitis. Mechanistic investigation revealed that the caspase-4/GSDMD/IL-1β non-canonical pyroptosis pathway was the major contributor to the pathogenesis of periodontitis. Through the construction of animal periodontitis models, we demonstrated that GSDMD-mediated PDLSC pyroptosis participated in periodontal tissue destruction and could be therapeutically targeted.

## Materials and Methods

### Study Participants

The patients in this study included 87 adults (43 females and 44 males, age 18–45 years) from the Department of Oral Surgery, Ninth People’s Hospital, Shanghai Jiao Tong University. All participants were asked to give their consent in written form after being informed of the purpose and protocol of the study. This study was approved by the Research Ethics Committee of the Ninth People’s Hospital (approval NO. SH9H-2020-TK60-1). Patients were divided based on clinical diagnosis into a severe periodontitis group and a healthy group. The patients in the severe periodontitis group (*n* = 16, 8 females and 8 males) were diagnosed by the following indices: clinical attachment level (CAL) ≥ 5 mm, alveolar crest bone loss (BL) > 6 mm without reaching the tooth apex, positive pulpal sensitivity, the necessity of tooth extraction and reach stage III with potential for additional tooth loss (Caton et al., 2018). The healthy group (*n* = 22, 11 females and 11 males) consisted of patients with impacted or partially erupted third molars and teeth requiring removal for orthodontic therapy.

### GCF and Periodontium Sampling

We obtained gingival crevicular fluid (GCF) from periodontitis patients (*n* = 65) and healthy patients (*n* = 10). For GCF collection, teeth were air dried and isolated with cotton rolls, supragingival plaque was removed, and then a collection strip was inserted into the sulcus for 1 min. Samples were eluted from the strip in 100 μl of phosphate-buffered saline (PBS) by centrifugation in centrifuge tubes. The eluates were stored at −80°C until assays were performed. Periodontium tissues were collected from 10 healthy (orthodontic) teeth and 10 teeth with severe periodontitis after extraction from patients. Periodontium tissues were isolated and prepared for protein extraction and western blot analysis. The antibodies used are given in Supplementary Table 1.

### Immunohistochemistry (IHC), Immunofluorescence (IF)

Human teeth were obtained from 6 patients with severe periodontitis and 6 healthy (orthodontic) patients. The teeth were fixed with 4% paraformaldehyde (PFA) for 24 h, decalcified with 10% EDTA (pH = 7.2) for 10 weeks and then sectioned in sections. The sections were incubated at 4°C overnight with primary antibodies, then washed with PBS and incubated with secondary antibody for 1 h. For IHC, sections were visualized with a Diaminobenzidine (DAB) Kit (MXB, China) and restained with hematoxylin. For IF, sections were counterstained with 4′,6-diamidino-2-phenylindole (DAPI). Images were taken with a high-resolution digital camera (Olympus DP 73, Japan).

### Cell Isolation, FACS Sorting and PDLSC Culture

Human periodontal ligament (PDL) tissues of third molars were obtained from 6 healthy donors (3 females and 3 males) undergoing tooth extraction for orthodontic reasons. Following the extraction, the PDL was minced into 1 mm^3^ pieces and digested for 30 min at 37°C in 0.3% Type I collagenase (Sigma, United States). After centrifugation, the precipitate was transferred to culture flasks with α-minimum essential medium (HyClone, United States) supplemented with 20% fetal bovine serum (Gibco, United States), 100 U/mL penicillin and 100 mg/mL streptomycin (HyClone, United States) and then cultured at 37°C in a 5% CO_2_ incubator. A total of 1 × 10^6^ cells were harvested, and the single-cell suspension was incubated with mesenchymal stem cell surface markers: FITC CD90, PerCP-Cy5.5 CD105, APC CD73, and PE CD44 (BD, United States) (Bartold and Gronthos, 2017; Al-Habib and Huang, 2019; Trubiani et al., 2019). PDLSCs were acquired immediately after staining using a FACSCalibur flow cytometer (BD, United States) equipped with the Cell Quest program (BD, United States). Then, PDLSCs were expanded with α-MEM containing 10% FBS and 1% antibiotics at 37°C in a 5% CO_2_ incubator.

### Osteogenic Differentiation

To induce osteoblast differentiation, a total of 1 × 10^5^ PDLSCs were seeded into each well of 6-well plates. Upon reaching a density of 70%, the PDLSCs were grown in osteogenic differentiation medium (Cyagen, United States) for 21 days. In some osteogenic induction assays, the PDLSCs were treated with recombinant human IL-1β protein (R&D, United States) at the indicated concentration every 2 days. After 21 days, the cells were fixed with 4% PFA in PBS and stained with 2% Alizarin red (pH = 4.2).

### Adipogenic Differentiation

For adipogenic induction, PDLSCs were plated in 6-well plates. When the PDLSCs reached 100% confluence, the growth medium was changed to adipogenic differentiation medium (Cyagen, United States), and the manufacturer’s protocol was followed. In some adipogenic induction assays, PDLSCs were treated with recombinant human IL-1β protein (R&D, United States) at the indicated concentration every 2 days. After 21 days, the cells were fixed in 4% PFA for 30 min and stained with Oil red O.

### Chondrogenic Differentiation

To induce chondrogenic differentiation, a total of 2 × 10^5^ PDLSCs were centrifuged in a 15 mL polypropylene tube (Corning, United States). Cell pellets were treated with chondrogenic differentiation medium (Cyagen, United States) according to the manufacturer’s protocol. After 30 days, pellets were fixed with 4% PFA and then made into paraffin sections. Chondrogenic differentiation was evaluated by staining with Alcian blue solution (Cyagen, United States).

### Osteoclast Differentiation and TRAP Staining

THP-1 (human monocyte) cells were purchased from the Shanghai Institute of Cell Biology, China. For osteoclast differentiation, 3 × 10^4^ THP-1 cells were seeded in each well of 48-well plates and cultured with osteoclast differentiation medium, according to the previous studies (Li et al., 2017; Ren et al., 2019). In some osteoclastogenesis assays, THP-1 cells were treated with recombinant human IL-1β protein (R&D, United States) at the indicated concentration every 2 days. After 14 days of induction, cells were fixed with 4% PFA and stained with TRAP staining solution (Jiancheng Biotech, China). TRAP-positive cells with three or more nuclei were considered osteoclasts, and the number of osteoclasts in each well was counted under a microscope.

### Bacterial Strains and Cell Stimulation

*Porphyromonas gingivalis* (*P. gingivalis*) strain ATCC 33,277 was obtained from the Shanghai Key Laboratory of Stomatology at Shanghai Jiao Tong University School of Medicine. *P. gingivalis* was grown on anaerobic blood agar plates with 80% N_2_, 10% H_2_, and 10% CO_2_ for 3–5 days. It was then inoculated into brain heart infusion broth supplemented with 5 μg/mL hemin and 1 μg/mL vitamin K for 24 h until reaching an optical density of 0.04 at 660 nm, corresponding to 10^8^ colony-forming units (CFU)/mL. The bacteria were washed and resuspended in α-MEM to infect the PDLSCs at multiplicities of infection (MOIs) of 1:10, 1:50, and 1:100 for 24 h. In some experiments, the PDLSCs were pretreated with Z-VAD-FMK (Sellect, United States), Z-YVAD-FMK (BioVision, United States), Z-LEVD-FMK (BioVision, United States) and DMSO (Sigma, United States) at the indicated concentrations for 1 h before bacterial infection.

### Stimulation of Canonical and Non-canonical Pyroptosis

To activate Caspase-4/11-mediated non-canonical pyroptosis, PDLSCs were transfected with 5 μg/mL LPS (Sigma, United States) by using 0.25% v/v FuGENE HD (Promega, United States). Activation of Caspase-1-mediated canonical pyroptosis: PDLSCs were pretreated with 50 ng/mL PMA (Sigma, United States) for 1 h and then stimulated with 10 μM nigericin (Sigma, United States) or 3 mM ATP (Sigma, United States) (Shi et al., 2015; Okondo et al., 2017; Wang et al., 2020). In some non-canonical pyroptosis activation experiments, the PDLSCs were pretreated with Z-VAD-FMK, Z-YVAD-FMK, Z-LEVD-FMK, and DMSO (Sigma, United States) at the indicated concentrations for 1 h.

### Transwell Coculture Assay

PDLSCs were seeded into the lower 6-well transwell chamber at 1 × 10^5^ cells per well and cultured to 80–90% confluence in growth medium. The medium was then replaced with osteogenic differentiation medium or adipogenic differentiation medium. PDLSCs were added to the upper chamber at 5 × 10^5^/well with LPS treatment, with LPS^FuGene^ treatment or without treatment. The cells remaining in the upper transwell chambers were replaced every 2 days. After 21 days of coculture in the differentiation medium, the cells in the lower chamber were harvested for further analysis.

THP-1 cells were plated at 3 × 10^4^ cells per well in the lower 48-well transwell chamber. The medium was then replaced with osteoclast differentiation medium. PDLSCs were added to the upper chamber at 1 × 10^4^/well with LPS treatment, with LPS^FuGene^ treatment or without treatment. The cells remaining in the upper transwell chambers were replaced every 2 days. After 14 days of coculture, the cells in the lower chamber were harvested for osteoclastogenesis analysis.

### Animal Studies

*Gsdmd*−/− mice were bred on a C57BL6/N background and provided by Jiangsu GemPharmatech Co., Ltd. (Nanjing, China). Sixteen male Sprague-Dawley (SD) rats (200–250 g, 8 weeks) were obtained from Shanghai Animal Experimental Center, China. In this study, all animals were maintained in a reserved facility and given free access to food and water under specific pathogen-free conditions. The animal welfare and experimental procedures were approved by the Animal Care and Use Committee of Shanghai Jiao Tong University Affiliated Ninth People’s Hospital (approval NO. SH9H-2020-A132-1). To construct the experimental periodontitis model, a 4-0 silk ligature was placed around the left first molar of the animal maxilla for 14 days under anesthesia with sodium pentobarbital (Bhattarai et al., 2016; Santana et al., 2016; Marchesan et al., 2018). The ligatures remained in place throughout the experimental period and were checked every 2 days to ensure subgingival placement. Sixteen SD rats were randomly divided into four groups (*n* = 6/group): sham, ligature, ligature + Caspase-4 inhibitor (Z-LEVD-FMK, 50 ng/μl), and ligature + IL-1β antibody (25 pg/μl). The drugs were administered into the subperiosteum at the left buccal and palatal gingivae of the first maxillary molars every 2 days in a volume of 20 μl for 14 days. Mice were divided into three groups (*n* = 6/group): wild type (WT) + sham, WT + ligature, and Gsdmd-knockout + ligature. On day 14, all animals in each group were sacrificed by carbon dioxide inhalation for further experimental analyses.

### Micro-Computed Tomography (Micro-CT) Imaging and Bone Parameter Analysis

All animals were anesthetized, and their maxillary specimens were scanned by a high-resolution micro-CT scanner SkyScan 1176 (Bruker, Belgium). The parameters used were 70 kV, 353 uA, and 360° rotations, with a 0.5 mm thick aluminum filter. The analyzer software CT Analyzer/CT Volume (Bruker, Belgium) was used for the visualization and quantification of 2D and 3D data. The distances from the cement-enamel junction (CEJ) to the alveolar bone crest (ABC) in the periodontitis-induced area in the images were measured to confirm alveolar bone loss and tissue damage. The bone mineral density (BMD, g/cm3) and bone/tissue volume (BV/TV, %) were calculated after the selection of a three-dimensional region of interest (ROI) around the first upper molar.

### Histological Analysis

After scanning by micro-CT, the maxilla specimens were fixed in 4% PFA for 24 h and decalcified in 10% EDTA solution for 5 weeks, and then sectioned in sections. The sections were mounted on glass slides and stained with hematoxylin and eosin (H&E). Histopathological changes in stained tissues were observed using an optical microscope (Olympus, Japan).

### RNA Extraction and Quantitative Real-Time PCR

Total RNA was extracted using RNAiso Plus (Takara, Japan) according to the manufacturer’s instructions. The PrimerScript^^TM^ RT Reagent Kit with gDNA Eraser (Takara, Japan) was used to synthesize first-strand cDNA from equivalent amounts of RNA. Then, quantitative real-time PCR was performed using SYBR Premix EX Taq^^TM^ (Takara, Japan) following a real-time PCR detection system (Roche, CH). Relative gene expression was normalized to the housekeeping gene *36B4* using the –ΔΔCt method. Primer sequences are summarized in Supplementary Table 2.

### LDH

LDH release was measured using a Cyto Tox 96 Non-Radioactive Cytotoxicity Assay Kit (Promega, United States) according to the manufacturer’s protocol. All values represent the percentage of LDH release compared with a maximum lysis control (1% Triton X-100-lysed cells).

### ELISA

The amount of IL-1β in gingival crevicular fluids and cell culture supernatants from different groups was measured by ELISA according to the manufacturer’s guidelines. A Human IL-1 beta/IL-1F2 Quantikine ELISA Kit (R&D, United States) was used.

### Statistical Analysis

All the results are shown as the mean ± SEM using GraphPad Prism version 8.01 (GraphPad Software, United States). The pairwise differences between two groups were evaluated for statistical significance using the unpaired Student’s *t*-test with Welch’s correction. For comparison of more than 2 groups, one-way ANOVA was used. *P*-values less than 0.05 were considered statistically significant (^∗^*P* < 0.05, ^∗∗^*P* < 0.01, ^∗∗∗^*P* < 0.001).

## Results

### Inflammatory Lesions in Periodontitis Are Correlated With Pyroptosis in PDLSCs

**FIGURE 1:**
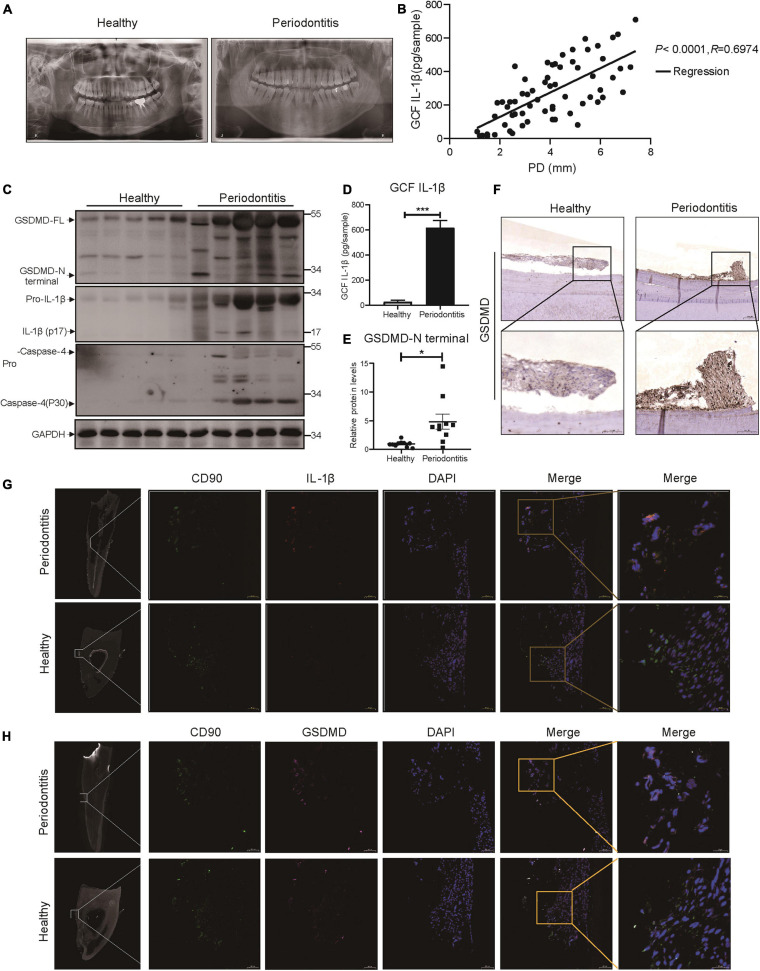
The inflammatory lesions in periodontitis were correlated with the pyroptosis of PDLSCs. **(A)** Panoramic radiograph from healthy and periodontitis patients. **(B)** Scatter plot and regression line for the regression between GCF IL-1b level and probing depth (PD) from periodontitis patients (*n* = 65). **(C)** Periodontium tissues were collected from periodontitis patients and healthy individuals, and the expression of GSDMD, IL-1β, and Caspase-4 was analyzed by western blot. GAPDH was used as the loading control. **(D)** GCF from healthy subjects and periodontitis patients was analyzed to determine the concentrations of human IL-1β by ELISA. **(E)** The intensities of the GSDMD-N terminus were quantified using imaging software and normalized to that of GAPDH. Ten periodontium tissue specimens from periodontitis patients and healthy individuals were examined. **(F)** Representative immunohistochemical staining of GSDMD in human periodontal ligament sections from healthy and periodontitis patients. The signals of GSDMD appear brown in sections counterstained with hematoxylin (blue). Experiments were repeated independently more than three times. **(G)** Immunofluorescence staining of CD90 (green) and IL-1β (red) in human tooth longitudinal sections from severe periodontitis patients and healthy (orthodontic) patients. **(H)** Immunofluorescence staining of CD90 (green) and GSDMD (purple) in human tooth longitudinal sections from severe periodontitis patients and healthy patients. Nuclei were identified by staining with DAPI. Scale bars, 50 μm. The data are presented as the mean ± SEM. *P < 0.05, ***P < 0.001.

We collected panoramic radiographs, gingival crevicular fluid (GCF), teeth and periodontal tissues from severe periodontitis patients and healthy patients. X-ray imaging combined with clinical examination demonstrated that periodontitis patients showed obvious alveolar bone resorption, gingival recession, and reduced periodontal tissue adhesion compared with healthy patients (Figure 1A). To look for the relationship between pyroptosis and periodontitis, first, a Pearson’s correlation analysis was conducted between GCF IL-1β level (IL-1β release was the typical event of pyroptosis) and probing depth (clinical index of periodontitis), which demonstrated a significant positive correlation in the two parameters (*P* < 0.0001, *R* = 0.6974) (Figure 1B). Consistently, the active form of IL-1β was highly expressed in periodontal tissues and GCFs from periodontitis patients (Figures 1C,D). We next detected the expression of GSDMD (the key executor of pyroptosis) in periodontal tissues from periodontitis patients. In support of this hypothesis, we found that the GSDMD level was also upregulated, accompanied by increased release of the GSDMD N-terminal fragment (Figures 1C,E). Additionally, strong immunostaining for GSDMD was observed in the periodontal ligaments from periodontitis patients (Figure 1F). As shown in Figure 1C, caspase-4 was also highly expressed in the periodontal tissues of periodontitis patients. These findings suggested that caspase-4 was activated in periodontitis tissues, leading to GSDMD cleavage and IL-1β release.

As previous works proposed that IL-1β was a useful indicator for evaluating the host response during periodontitis and that human periodontal ligament cells released IL-1β under cyclic stretch (Zhuang et al., 2019; Aral et al., 2020), we detected the localization of IL-1β in periodontal ligament from periodontitis patients. Interestingly, immunofluorescence analysis showed that PDLSCs (CD90+) were the source of IL-1β production and the amount of PDLSCs was decreased in periodontitis patients (Figure 1G), which indicated the occurrence of pyroptosis in PDLSCs during periodontitis. To further prove PDLSC was the cell type that pyroptosis happened in periodontitis, we also examined the localization pattern of GSDMD. The co-staining results demonstrated that GSDMD was enriched in PDLSCs in periodontitis patients (Figure 1H). Taken together, the above results suggest that periodontitis primarily triggers the production of inflammatory cytokines by inducing PDLSC pyroptosis, which in turn has an important role in the pathogenesis of periodontitis.

### *P. gingivalis* Induces PDLSC Pyroptosis Through Caspase-4 Activation

We used a flow cytometer to acquire PDLSCs based on surface molecules. PDLSCs positively expressed MSC markers, including CD90, CD44, CD105, and CD73 and were negative for hematopoietic progenitor cell surface markers (CD34, CD11b, CD19, CD45, and HLA-DR) (Supplementary Figure 1A). PDLSCs were grown in osteogenic, adipogenic and chondrogenic induction media for 21 or 30 days and assessed. Cells displayed calcium deposition positively stained by Alizarin red, cytoplasmic lipid accumulation positively stained by Oil red O, and acidic polysaccharide extracellular matrices positively stained by Alcian blue (Supplementary Figures 1B–D). Furthermore, the protein levels of osteogenic markers (ALP, RUNX2, and OPN) and adipogenic markers (APN, PPAR-γ, and C/EBP-α) were both significantly elevated under osteogenic or adipogenic differentiation (Supplementary Figures 1E,F). All of these results demonstrated that PDLSCs are stem cells of mesenchymal sources with powerful multipotency.

**FIGURE 2:**
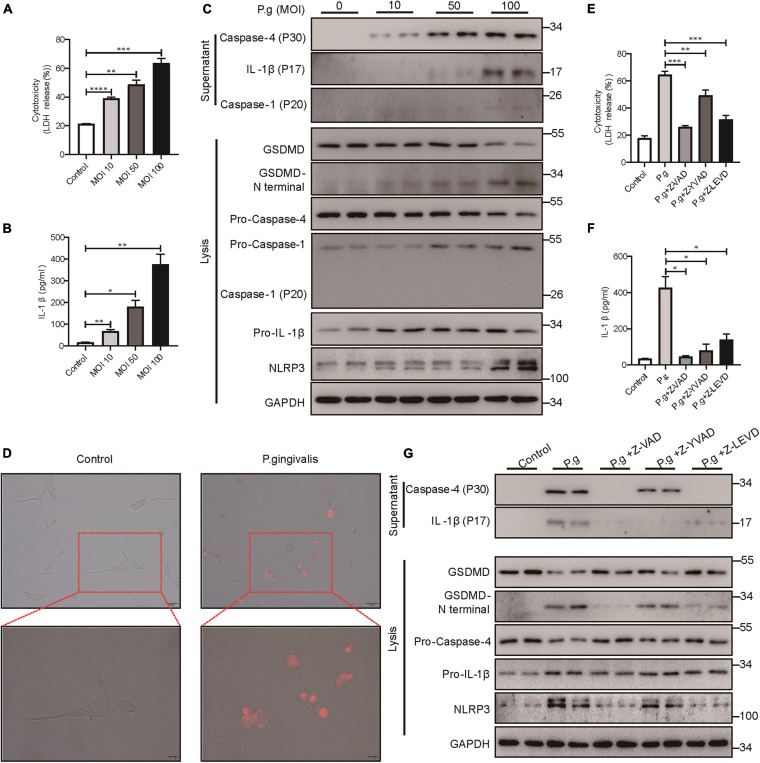
*P. gingivalis* induced pyroptosis in PDLSCs dependent on caspase-4. **(A–C)** PDLSCs were infected with *P. gingivalis* (MOI, 10, 50, or 100) for 24 h. **(A)** The amount of cytoplasmic LDH released into the culture supernatant was measured by an LDH Cytotoxicity Assay Kit. **(B)** Cell culture supernatants were assayed for human IL-1β by ELISA. **(C)** The IL-1β, Caspase-4 and Caspase-1 secreted into the culture supernatants and the GSDMD cleavage, Pro-IL-1β, Pro-Caspase-4, Pro-Caspase-1, NLRP3, and GAPDH in the cell lysates (lysis) were detected by immunoblotting. **(D)** The morphology of PDLSCs with *P. gingivalis* (MOI, 100) infection. Cells were stained with propidium iodide (PI, red) and analyzed under a microscope. **(E–G)** PDLSCs were pretreated with the pan-caspase inhibitor Z-VAD-FMK (20 μM), the caspase-1 inhibitor Z-YVAD-FMK (20 μM), or the capase-4 inhibitor Z-LEVD-FMK (20 μM) for 1 h before *P. gingivalis* (MOI, 100) infection for 24 h. **(E,F)** Cell culture supernatant was collected and assayed for LDH release **(E)** and IL-1β secretion **(F)**. **(G)** The IL-1β and Caspase-4 secreted into the culture supernatants and the GSDMD cleavage, Pro-IL-1β, Pro-Caspase-4, NLRP3, and GAPDH in the cell lysates (lysis) were detected by immunoblotting. The data are presented as the mean ± SEM. *P < 0.05, **P < 0.01, ***P < 0.001, ****P < 0.0001.

Periodontitis is a chronic inflammatory disease caused by major periodontopathogens. To determine whether PDLSCs undergo pyroptosis during periodontitis, we used *P. gingivalis* to mimic the pathogenetic process of periodontitis. We found that PDLSCs infected with *P. gingivalis* presented robust lactate dehydrogenase (LDH) release and increased IL-1β release, which were both gradually elevated in an MOI-dependent manner (Figures 2A,B). *P. gingivalis* activated caspase-4, and the amount of processed caspase-4 subunit in the culture supernatants was increased depending on the MOI of *P. gingivalis* (Figure 2C). In addition to caspase-4 activation, mature IL-1β release and GSDMD cleavage (indicators of terminal pyroptosis events) were both significantly increased under *P. gingivalis* infection (Figure 2C). In addition, the amount of activated caspase-1 did not show a marked increase in the culture supernatants compared with caspase-4 (Figure 2C). Furthermore, PDLSCs infected with MOI 100 *P. gingivalis* developed the typical pyroptotic morphology with cell swelling, membrane blebbing and propidium iodide (PI) uptake (Figure 2D). These results suggested that *P. gingivalis* promotes caspase-4 activation, GSDMD cleavage and mature IL-1β release.

To determine whether *P. gingivalis*-induced PDLSC pyroptosis was dependent on caspase-4 activation, cells were pretreated with the caspase pan inhibitor (Z-VAD-FMK), caspase-1 specific inhibitor (Z-YVAD-FMK) or caspase-4 specific inhibitor (Z-LEVD-FMK) prior to *P. gingivalis* challenge. As shown in Figures 2E–G, caspase pan inhibitor or caspase-4 specific inhibitor alleviated LDH release, IL-1β release and GSDMD cleavage, indicating that *P. gingivalis*-induced PDLSC pyroptosis was dependent on caspase-4 activation instead of caspase-1. Moreover, consistent with the important role of caspase-1 in IL-1β maturation (Monteleone et al., 2018), pretreatment with a caspase-1-specific inhibitor blocked the conversion of pro-IL-1β into its biologically active form IL-1β in response to *P. gingivalis* infection (Figures 2F,G).

### The Caspase-4-Mediated Non-canonical Pathway Dominantly Contributes to PDLSC Pyroptosis

**FIGURE 3:**
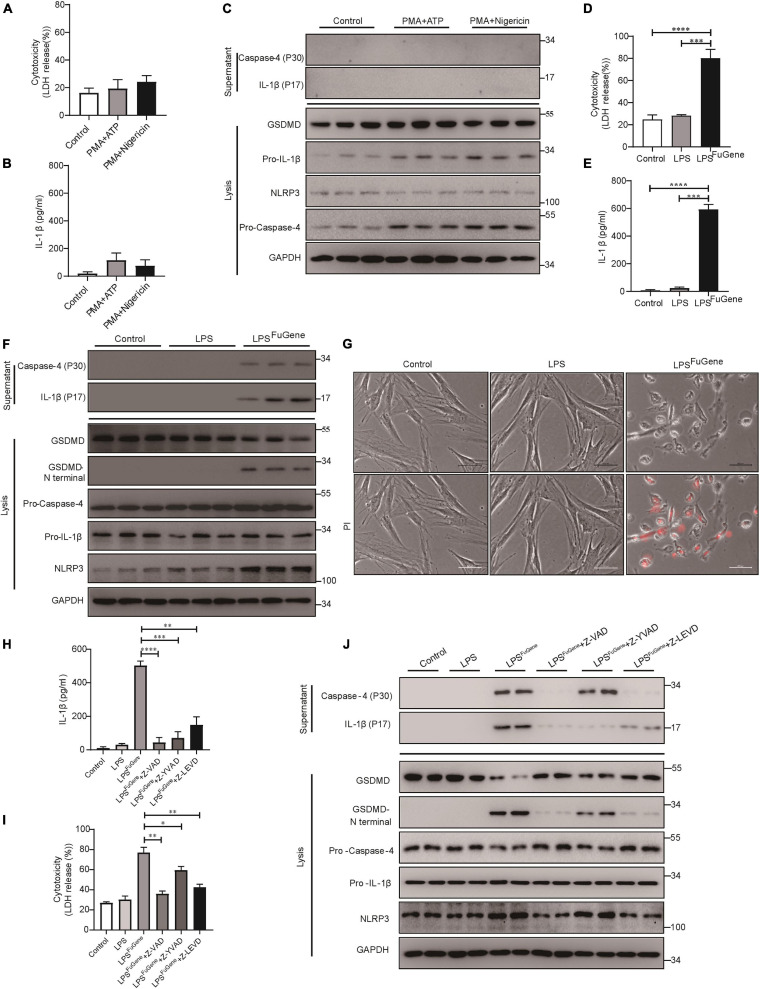
PDLSC pyroptosis was mediated by the caspase-4-dependent non-canonical pyroptosis pathway. **(A–C)** PDLSCs were pretreated with PMA for 1 h and stimulated with ATP or nigericin for 4 h. **(A,B)** Cell culture supernatant was collected and assayed for LDH release **(A)** and IL-1β secretion **(B)**. **(C)** The IL-1β and Caspase-4 secreted into the culture supernatants and the GSDMD cleavage, Pro-IL-1β, Pro-Caspase-4, NLRP3, and GAPDH in the cell lysates (lysis) were detected by immunoblotting. **(D–G)** PDLSCs were treated with LPS or transfected with LPS using FuGene HD for 16 h. Cells without treatment were used as controls. **(D,E)** Cell culture supernatant was collected and assayed for LDH release **(D)** and IL-1β secretion **(E)**. **(F)** The IL-1β and Caspase-4 secreted into the culture supernatants and the GSDMD cleavage, Pro-IL-1β, Pro-Caspase-4, NLRP3, and GAPDH in the cell lysates (lysis) were detected by immunoblotting. **(G)** Representative images of propidium iodide (PI, red) uptake. **(H–J)** PDLSCs were pretreated with the pan-caspase inhibitor Z-VAD-FMK (20 μM), the caspase-1 inhibitor Z-YVAD-FMK (20 μM), or the capase-4 inhibitor Z-LEVD-FMK (20 μM) for 1 h before transfection with LPS (LPS^FuGene^) for 16 h. **(H)** IL-1β ELISA in supernatants. **(I)** Percentage of LDH release in supernatants. **(J)** The IL-1β and Caspase-4 secreted into the culture supernatants and the GSDMD cleavage, Pro-IL-1β, Pro-Caspase-4, NLRP3, and GAPDH in the cell lysates (lysis) were detected by immunoblotting. The data are presented as the mean ± SEM. *P < 0.05, **P < 0.01, ***P < 0.001, ****P < 0.0001.

Next, to further investigate the mechanism of PDLSC pyroptosis, both canonical pyroptosis and non-canonical pyroptosis were triggered *in vitro*. When canonical pyroptosis was triggered by PMA plus ATP or LPS plus nigericin in PDLSCs, the release of LDH and IL-1β did not show any significant change in the culture supernatants (Figures 3A,B). In addition, we did not detect any cleavage of GSDMD or the active form of IL-1β during canonical pyroptosis activation (Figure 3C). In contrast, when LPS (the major pathogenic factor of gram-negative bacteria) was transfected into PDLSCs to activate the non-canonical pyroptosis pathway, we found that the amounts of LDH and IL-1β in the culture supernatants were both significantly increased (Figures 3D,E). Consistent with the *P. gingivalis* infection, LPS-transfected PDLSCs also showed the activation of caspase-4, along with the cleavage of GSDMD and release of mature IL-1β (Figure 3F). Moreover, after LPS transfection, PDLSCs exhibited signature morphology of pyroptosis with cell swelling and rupture of the membrane (Figure 3G). To determine whether cytoplasmic LPS-induced non-canonical pyroptosis in PDLSCs was also dependent on caspase-4 activation, cells were pretreated with caspase inhibitors. Similar to the inhibitory effect of Z-LEVD-FMK in *P. gingivalis*-induced PDLSC pyroptosis, pretreatment with the caspase-4-specific inhibitor alleviated LDH release, GSDMD cleavage and mature IL-1β release in response to intracellular LPS stimulation (Figures 3H–J). Collectively, these data demonstrated that the caspase-4-mediated non-canonical pyroptosis pathway plays a dominant role in PDLSC pyroptosis.

### PDLSC Pyroptosis Inhibits Osteoblast Differentiation and Promotes Osteoclast Differentiation

**FIGURE 4:**
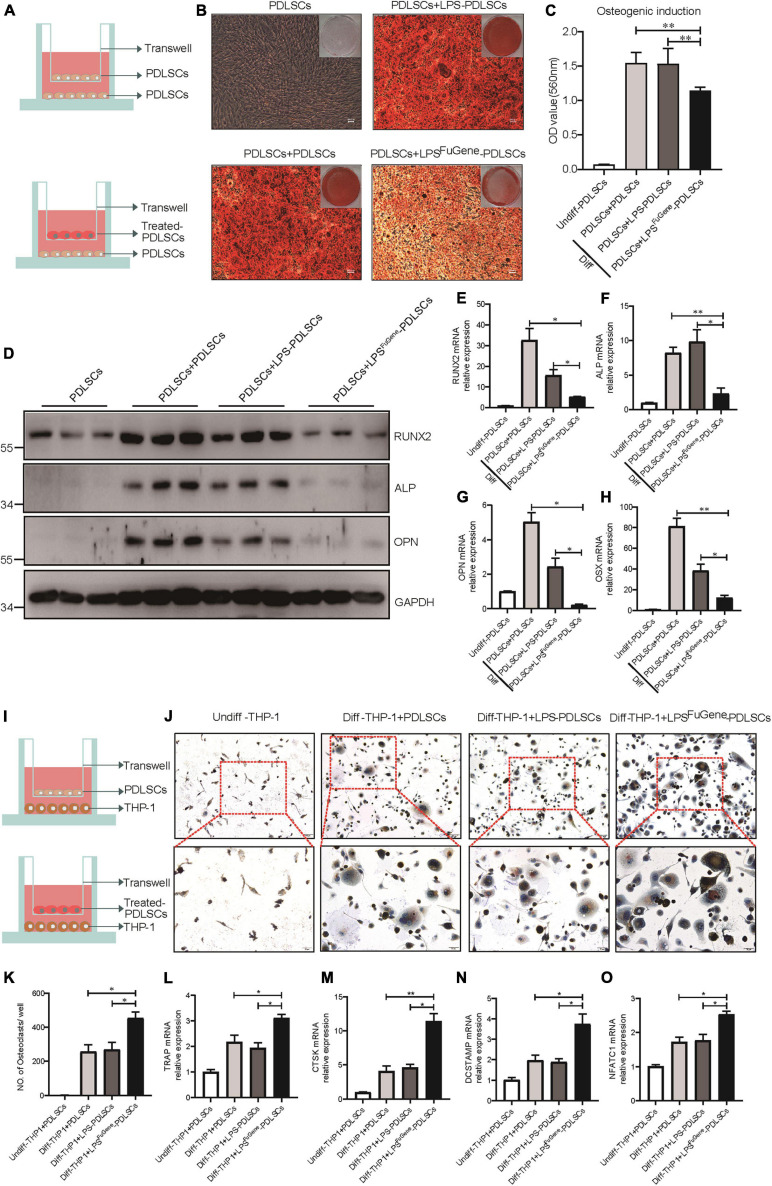
The paracrine effect of PDLSC pyroptosis on osteoblast differentiation and osteoclast differentiation. **(A)** Schematic diagram of coculture design indicating the placement of PDLSCs with LPS, with LPS^FuGene^ treatment, or without treatment on the transwell insert with the lower chamber containing healthy PDLSCs. **(B–H)** Cells in the cocultured transwell system were maintained in osteogenic differentiation medium for 21 days. PDLSCs in a single culture (without coculture) were maintained in MEM-α medium as a control. **(B)** Entire plate views and micrographs of alizarin red staining after culturing in osteogenic differentiation medium. **(C)** Quantification of the alizarin red staining results. **(D)** Immunoblots for RUNX2, ALP, and OPN in whole-cell lysates. GAPDH was used as a loading control. *RUNX2* **(E)**, *ALP* **(F)**, *OPN* **(G)**, and *OSX* **(H)** mRNAs were subjected to real-time PCR analysis, and the expression levels were normalized to that of *36B4*. **(I)** Schematic diagram of coculture design indicating the placement of PDLSCs with LPS treatment, with LPS^FuGene^ treatment, or without treatment on transwell insert with the lower chamber containing THP-1 cells. **(J–O)** The THP-1 cells in the coculture system were cultured in osteoclast differentiation medium for 14 days. THP-1 cells in a single culture were maintained in RPMI-1640 medium as a control. **(J)** Micrographs of TRAP staining after culturing in osteoclast differentiation medium. **(K)** Quantification of TRAP-positive multinucleated cells (nuclei > 3). *TRAP* **(L)**, *CTSK* **(M)**, *DCSTAMP* **(N)**, and *NFATC1* **(O)** mRNAs were subjected to real-time PCR analysis, and the expression levels were normalized to that of *36B4*. The data are presented as the mean ± SEM. *P < 0.05, **P < 0.01.

Previous works have demonstrated that the alveolar bone resorption of periodontitis was due to an imbalance between osteogenic differentiation and osteoclastogenic differentiation (Hienz et al., 2015), with increased osteoclasts and decreased osteoblasts. To evaluate whether pyroptotic PDLSCs affected the osteogenic differentiation of healthy PDLSCs (hPDLSCs), they were cocultured in osteogenic differentiation medium (Figure 4A). PDLSCs with LPS^FuGene^-induced pyroptosis were seeded in the upper transwell chambers and replaced every 2 days. After 21 days of coculture, we subjected the lower chambers (hPDLSCs under osteogenic induction) to Alizarin red staining. A statistically significant decline in mineralization levels was found in hPDLSCs cocultured with pyroptotic PDLSCs, while hPDLSCs cocultured with empty or LPS-treated PDLSCs displayed no significant differences (Figures 4B,C). Additionally, the protein levels of the osteoblast markers ALP, RUNX2, and OPN were all significantly decreased in hPDLSCs cocultured with pyroptotic PDLSCs, and the transcription of these osteoblast markers was downregulated (Figures 4D–H). Similar to osteogenic differentiation, hPDLSCs cocultured with pyroptotic PDLSCs showed obvious inhibition of adipogenic differentiation, with decreased lipid droplet formation and downregulation of adipogenic markers (Supplementary Figure 2). The above data suggested an inhibitory effect of pyroptotic PDLSCs on the multipotential differentiation of hPDLSCs, which may reduce the regeneration of damaged periodontal tissues.

We used a coculture system of pyroptotic PDLSCs and THP-1 (human monocyte) cells to further investigate the functional role of pyroptotic PDLSCs in osteoclastogenesis (Figure 4I). Cells were cocultured with medium supplemented with RANKL and M-CSF for 14 days. Following removal of the Transwell inserts, the lower chambers consisting of THP-1 cells were subjected to TRAP staining to assess osteoclastogenic differentiation. While THP-1 cells cocultured with empty or LPS-treated PDLSCs induced abundant TRAP-positive multinucleated osteoclasts, THP-1 cells cocultured with pyroptotic PDLSCs induced significantly more multinucleated osteoclasts (Figures 4J,K). The mRNA expression levels of the osteoclast markers *TRAP*, *CTSK*, *DCSTAMP*, and *NFATC1* were also found to be significantly elevated in THP-1 cells cocultured with pyroptotic PDLSCs (Figures 4L–O). These results indicated the promotion effect of pyroptotic PDLSCs on osteoclastogenic differentiation, which may aggravate the destruction resulting from periodontitis.

### PDLSC Pyroptosis-Mediated IL-1β Production Enhances Osteoclast Differentiation

**FIGURE 5:**
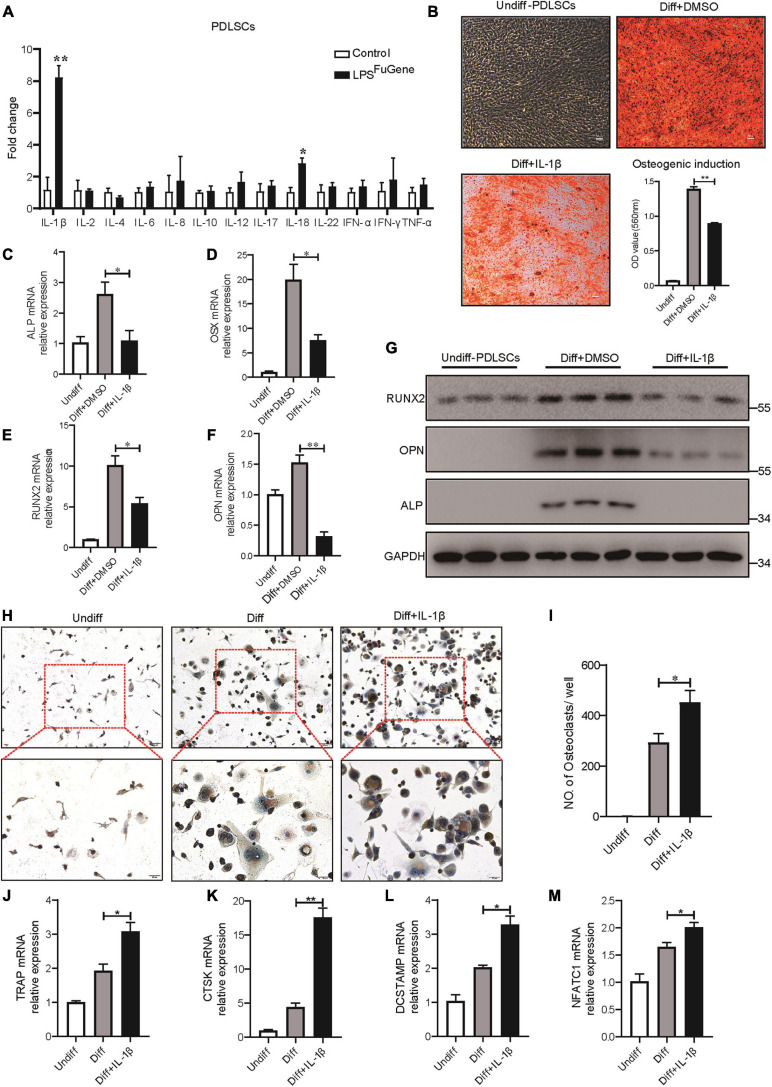
IL-1β was the key factor in PDLSC pyroptosis to regulate osteoblast and osteoclast differentiation. **(A)** Expression profiles of several inflammatory cytokines in PDLSCs under LPS^FuGene^ treatment for 16 h. **(B–G)** PDLSCs were incubated with osteogenic differentiation medium for 21 days with or without 500 pg/mL IL-1β treatment. **(B)** Representative images of alizarin red staining and quantification of the staining results. *ALP* **(C)**, *OSX* **(D)**, *RUNX2* **(E)**, and *OPN* **(F)** mRNAs were subjected to real-time PCR analysis, and the expression levels were normalized to that of *36B4*. **(G)** Immunoblots for RUNX2, ALP, and OPN in whole-cell lysates. GAPDH was used as a loading control. **(H–M)** THP-1 cells were incubated with osteoclast differentiation medium for 14 days with or without 500 pg/mL IL-1β treatment. **(H)** Micrographs of TRAP staining after culturing in osteoclast differentiation medium. **(I)** Quantification of TRAP-positive multinucleated cells (nuclei > 3). *TRAP* **(J)**, *CTSK* **(K)**, *DCSTAMP* **(L)**, and *NFATC1* **(M)** mRNAs were subjected to real-time PCR analysis, and the expression levels were normalized to that of *36B4*. The data are presented as the mean ± SEM. *P < 0.05, **P < 0.01.

As the most significant increase in IL-1β expression was detectable in the pyroptotic PDLSCs (Figure 5A), we examined whether the regulatory effect of PDLSC pyroptosis on osteogenic differentiation and osteoclastogenic differentiation was mediated by mature IL-1β release. Indeed, when the osteogenic differentiation of PDLSCs was induced by culturing in osteogenic media with or without recombinant human IL-1β protein, the osteogenic differentiation of PDLSCs was clearly inhibited by IL-1β treatment. This was evidenced by declining mineralization levels, decreased protein expression of osteoblast markers ALP, RUNX2, and OPN, and downregulation of the transcription of these osteoblast markers (Figures 5B–G). Analogously, the adipogenic differentiation of PDLSCs was significantly inhibited by IL-1β treatment (Supplementary Figure 3). The ability of PDLSCs to undergo osteogenic differentiation was greatly impaired by the addition of IL-1β protein, whereas the IL-1β antibody was able to rescue the inflammation-induced reduction in osteogenesis (Supplementary Figure 4A).

To test whether IL-1β also contributed to osteoclastogenesis, THP-1 cells were cultured in osteoclastogenic differentiation medium with or without IL-1β. After 14 days of IL-1β incubation, a marked increase in the number of multinucleated osteoclasts was observed through TRAP staining (Figures 5H,I). Furthermore, the expression levels of osteoclastogenic differentiation marker genes, such as *TRAP*, *CTSK*, *DCSTAMP*, and *NFATC1*, were significantly increased under IL-1β treatment (Figures 5J–M). When IL-1β antibody was added, the number of TRAP-positive multinucleated osteoclasts was markedly reduced (Supplementary Figure 4B). In brief, these results revealed that the regulatory effect of PDLSC pyroptosis on osteoblast and osteoclast differentiation was mediated by mature IL-1β release.

### Caspase-4-Mediated Pyroptosis Promotes the Progression of Ligature-Induced Rat Periodontitis

**FIGURE 6:**
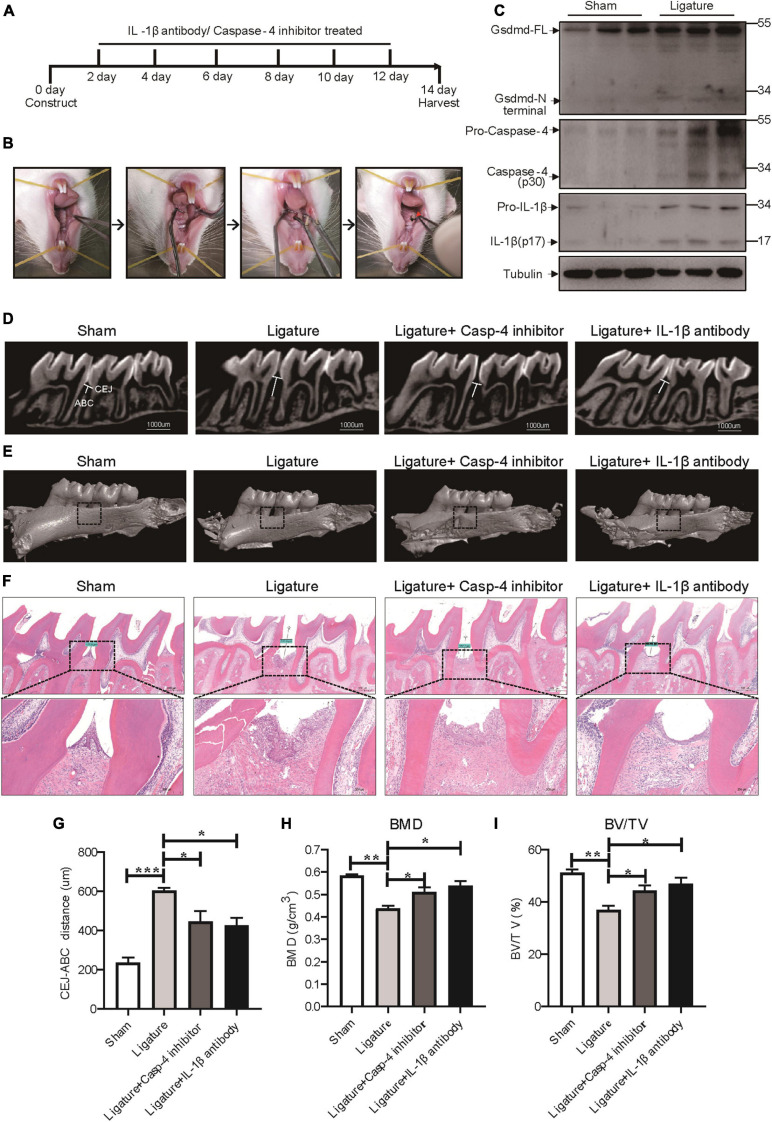
Caspase-4-mediated non-canonical pyroptosis was involved in ligature-induced rat periodontitis. **(A–I)** Rats were divided randomly into a sham group, ligature-induced rat periodontitis group (ligature group), ligature with caspase-4 inhibitor injection into the subperiosteum at the left buccal and palatal gingivae of the first maxillary molars group (Ligature + Casp-4 inhibitor group), and ligature with IL-1b antibody injection into the subperiosteum at the first maxillary molar group (Ligature + IL-1b antibody group). **(A)** Timeline of the animal experiment. **(B)** Technical procedures of ligature-induced rat periodontitis. A 4-0 silk ligature was looped around the first molar of the rat maxilla under anesthesia and maintained for 14 days. The red arrow indicated the drugs injection site. **(C)** Periodontium tissues were collected from ligature-induced rat periodontitis group and sham group, and the expression of Gsdmd, IL-1β, and caspase-4 was analyzed by western blot. Tubulin was used as the loading control. **(D)** Representative micro-CT sagittal images of maxillary molars after insertion of ligature. CEJ, cement-enamel junction; ABC, alveolar bone crest. The line indicates the distance from the CEJ to the ABC on the buccal side of the ligature site. **(E)** The images show the reconstructed sagittal 3D images from the computerized tomography of the maxillary molars. The squares formed by the continuous dotted line show visual differences in alveolar bone from different animal groups. **(F)** Tissue sections from rats were prepared after 14 days of periodontitis induction and processed for hematoxylin and eosin (H&E) staining. **(G)** The distances (um) between CEJ and ABC were measured after periodontitis induction. The bone mineral density **(H)** and bone/tissue volume **(I)** of the selected squares regions on the ligature site were calculated. The data are presented as the mean ± SEM. *P < 0.05, **P < 0.01, ***P < 0.001.

To extend our *in vitro* findings, we next investigated the role of caspase-4-mediated non-canonical pyroptosis in ligature-induced rat periodontitis. A periodontitis rat model was generated by ligatures around the first maxillary molar and then treated with caspase-4 specific inhibitor (Z-LEVD-FMK) or IL-1β antibody for 14 days (Figures 6A,B). Similar to human periodontal tissues, Gsdmd cleavage, mature IL-1β release and caspase-4 activation were both significantly increased in ligature-induced rat periodontitis (Figure 6C). Representative sagittal 3D and bidimensional views of the rat maxillary molars from each group using micro-CT scanning are shown in Figures 6D,E. Compared with the sham group, the ligature group showed a greater distance between the cement-enamel junction (CEJ) and alveolar bone crest (ABC). However, administration of caspase-4 inhibitor or IL-1β antibody significantly inhibited periodontal destruction, showing a decreased CEJ-ABC distance (Figures 6D,G). Consistently, the bone mineral density (BMD, g/cm3) and bone/tissue volume (BV/TV, %) values in alveolar bone from the ligature group were both significantly lower than the corresponding values in the sham group, whereas the ligature-induced damage to BMD and BV/TV was rescued by treatment with caspase-4 inhibitor or IL-1β antibody (Figures 6E,H,I). Additionally, prominent loss of alveolar bone, periodontal ligament damage and infiltration of inflammatory cells were observed in ligatured rats, while treatment with caspase-4 inhibitor or IL-1β antibody showed improved periodontal conditions compared with the ligature group (Figure 6F). Collectively, either inhibiting the activation of caspase-4 or blocking the function of IL-1β can attenuate periodontitis-mediated tissue damage, which indicates the critical role of caspase-4-mediated non-canonical pyroptosis in rat periodontitis.

### Gsdmd Deficiency Reduces Periodontal Inflammation and Bone Loss in a Mouse Model of Periodontitis

**FIGURE 7:**
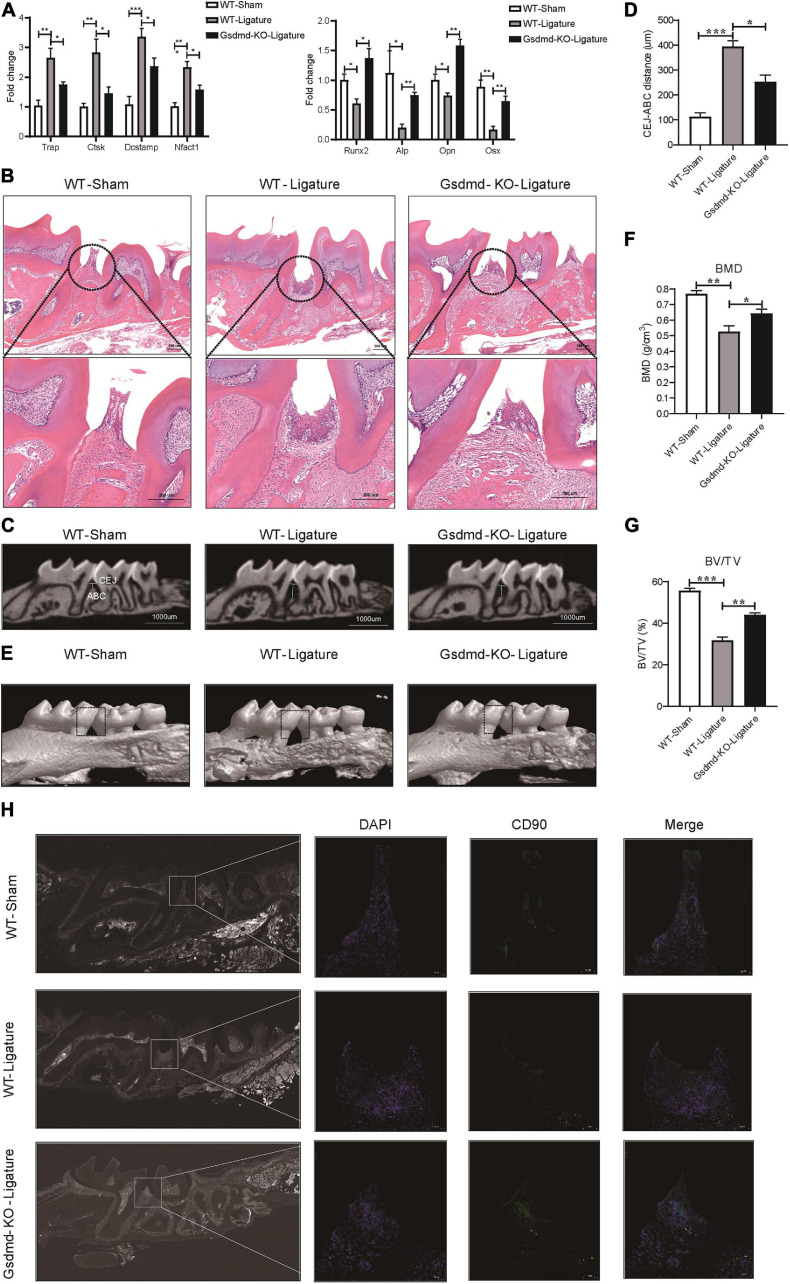
*Gsdmd*–/– mice presented with reduced periodontal inflammation and bone loss in experimental periodontitis. **(A–H)** Mice were divided into the WT-sham group, WT-ligature group, and Gsdmd-KO-ligature group. Silk thread was used to form a ligature around the left maxillary first molar for 2 weeks to induce periodontitis. **(A)** Periodontium tissues were collected from each group, and the mRNA expression of osteoblast markers (including *Runx2*, *Alp*, *Opn*, and *Osx*) and osteoclast markers (including *Trap*, *Ctsk*, *Dcstamp*, and *Nfact1*) was analyzed by real-time PCR. *36b4* was used as an internal control. **(B)** Tissue sections from mice were prepared after 14 days of periodontitis induction and processed for hematoxylin and eosin (H&E) staining. **(C)** Representative micro-CT sagittal images of maxillary molars after insertion of ligature. CEJ, cement-enamel junction; ABC, alveolar bone crest. The line indicates the distance from the CEJ to the ABC on the buccal side of the ligature site. **(D)** The distances (μm) between CEJ and ABC were measured after periodontitis induction. **(E)** The images of bone surrounding first molars were analyzed by 3D micro-computed tomography. The squares formed by the continuous dotted line show visual differences in alveolar bone from animal groups. The bone mineral density **(F)** and bone/tissue volume **(G)** of the selected square regions on the ligature site were calculated. **(H)** Immunofluorescence staining of CD90 (green) in mouse maxillary molar longitudinal sections from the periodontitis model and sham group. Nuclei were identified by staining with DAPI. Scale bars, 50 μm. The data are presented as the mean ± SEM. *P < 0.05, **P < 0.01, ***P < 0.001.

**FIGURE 8:**
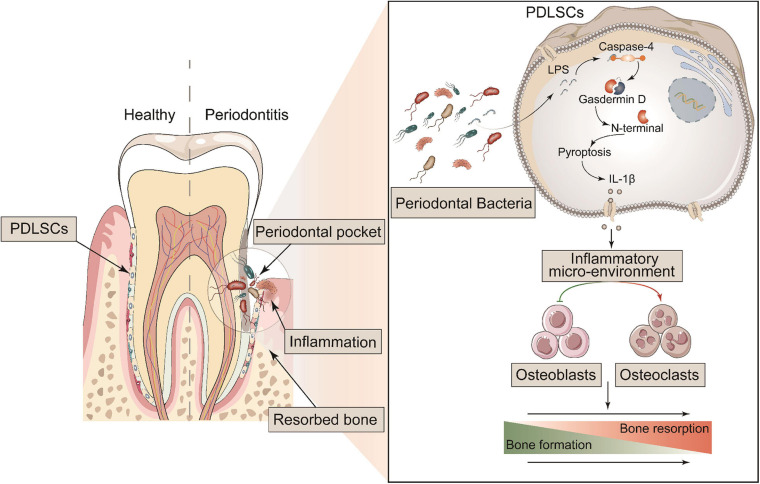
Schematic diagram of the mechanism of GSDMD-mediated PDLSC pyroptosis in the promotion of periodontitis. PDLSCs commit Caspase-4/GSDMD-mediated non-canonical pyroptosis in periodontopathogen-induced periodontitis. The pyroptotic PDLSCs express IL-1β and release it to the cellular microenvironment following membrane rupture. IL-1β disrupts the homeostatic balance of bone formation and resorption by inhibiting osteoblastogenesis and promoting osteoclastogenesis, jointly aggravating the process of periodontitis.

GSDMD, the key executor of pyroptosis, is cleaved by caspase-4 to mediate IL-1β release (Kayagaki et al., 2015). To better characterize the Caspase-4/GSDMD/IL-1β axis-mediated non-canonical pyroptosis as a potential driver of periodontitis, a mouse model with *Gsdmd* gene deficiency was used (Supplementary Figure 5A). In this model, the third codon of the *Gsdmd* gene was deleted by CRISPT/Cas-9 technology, and *Gsdmd*^–/–^ mice were confirmed by genotyping and western blot (Supplementary Figures 5B–D). After 14 days of periodontitis induction, the WT-ligature mice showed an increased expression of osteoclast markers *Trap*, *Ctsk*, *Dcstamp*, and *Nfatc1* and decreased expression of osteoblast markers *Runx2*, *Alp*, *Opn*, and *Osx*, compared with the WT-sham group. Conversely, the *Gsdmd*^–/–^ mice demonstrated fewer osteoclast markers expression and more osteoblast markers expression (Figure 7A). Histological analysis by H&E staining revealed that the WT-ligature mice showed a loss of alveolar bone, inflammatory cell infiltration and periodontal ligament damage after periodontitis induction, whereas *Gsdmd*^–/–^ mice had attenuated periodontitis-mediated tissue damage (Figure 7B). We assessed bone loss by measuring CEJ-ABC distances and found that WT-ligament mice showed significant bone loss, but this loss was alleviated in *Gsdmd*^–/–^ mice (Figures 7C,D). Meanwhile, BMD and BV/TV values were significantly decreased in the region between the first and second upper molars of WT-ligature mice compared with those of WT-sham mice. In contrast, the loss of BMD and BV/TV was attenuated significantly in *Gsdmd*^–/–^ mice under periodontitis induction (Figures 7E–G). Furthermore, prominent loss of PDLSCs (CD90^+^) was observed in WT-ligature mice, while Gsdmd-deficient mice demonstrated a rescue effect on PDLSC damage (Figure 7H). In brief, these data demonstrated that the genetic deletion of *Gsdmd* alleviated the loss of PDLSCs during experimental periodontitis and conferred protection from periodontal inflammatory bone loss. Therefore, GSDMD-mediated PDLSC pyroptosis was found to have a major contribution to periodontal inflammation and bone loss in this inflammatory disease, which supported a focus on PDLSC pyroptosis-targeted therapeutic approaches (Figure 8).

## Discussion

Periodontitis, the major cause of adult tooth loss, is an inflammatory disease caused by oral bacterial infection (Chen et al., 2020). Recently, new links have been established between oral microbe-induced pyroptosis and periodontitis. Pyroptosis is an important event in inflammatory processes and rapidly releases large amounts of activated cytokines, such as IL-1β, into the extracellular space (Kesavardhana et al., 2020). In this study, we provided a statistical correlation between IL-1β levels and the severity of periodontitis, which was consistent with previous reports that IL-1β in saliva or gingival crevicular fluid is a potential biomarker for periodontal disease (Gomes et al., 2016). Furthermore, GSDMD, the key executor of pyroptosis, was found to be highly expressed and activated in periodontitis tissues, and we reported for the first time that PDLSCs (CD90^+^) were the source of IL-1β production in periodontitis patients. These data suggested that IL-1β release in GSDMD-mediated PDLSC pyroptosis played an important role in the process of periodontitis.

The molecular mechanisms of GSDMD-mediated PDLSC pyroptosis were subsequently investigated. Although the relationship between oral bacteria and periodontal destructive inflammation was already well documented clinically (Hasan and Palmer, 2014), the mechanisms of periodontopathogen-induced pathogenic inflammation in periodontitis were poorly demonstrated. *P. gingivalis*, an oral gram-negative anaerobic bacterium, was primarily found in deep periodontal pockets, especially in sites with active disease (Kuula et al., 2009). We infected PDLSCs with *P. gingivalis* to mimic the pathogenetic process of periodontitis and found typical pyroptosis morphology of PDLSCs with cell swelling and massive release of cellular contents. *P. gingivalis* stimulated the release of the cleaved GSDMD-N terminal domain and active IL-1β in an MOI-dependent manner and increased the activation of caspase-4. The inhibition of caspase-4 significantly decreased *P. gingivalis*-induced GSDMD cleavage and IL-1β secretion in PDLSCs, suggesting that *P. gingivalis*-induced PDLSC pyroptosis was mediated by caspase-4 activation. Furthermore, canonical pyroptosis and non-canonical pyroptosis were triggered based on previous works to investigate the preferential pyroptosis pathway in periodontitis (Kayagaki et al., 2015; Chen X. et al., 2016). These *in vitro* experiments revealed that the caspase-4/GSDMD/IL-1β axis-mediated non-canonical pyroptosis pathway dominantly contributed to PDLSC pyroptosis.

Currently, the standard treatment of periodontitis focuses on the removal of periodontitis-associated microbial communities and inflammatory tissues by means of mechanical dental debridement, and PDLSCs have been proposed and used in periodontal regeneration (American Academy of Periodontology, 2011; Sanz et al., 2012; Vandana et al., 2015; Tassi et al., 2017). However, the outcomes of clinical stem cell-based therapies did not achieve the desired outcome, most likely due to the local inflammatory microenvironment of the diseased periodontium (Fawzy El-Sayed et al., 2019; Jia et al., 2020). Indeed, we found that the differentiation potential of PDLSCs was significantly decreased in a pyroptosis-induced inflammatory microenvironment. These findings suggested that the paracrine effects of pyroptotic PDLSCs were responsible for the low efficiency of periodontium regeneration. IL-1β had previously been shown to be an important proinflammatory cytokine that drove the pathogenesis of periodontitis and often amplified the effect of other cytokines (Papathanasiou et al., 2020). Some studies reported that IL-1β inhibited the osteogenesis of MSCs through the NF-κB and MAPK signaling pathway and exacerbated the destruction of alveolar bone by promoting osteoclast formation and activity (Mao et al., 2016; Aral et al., 2020). Consistently, IL-1β is the major inflammatory cytokine released in pyroptosis, and we found that it performed bidirectional regulation of osteogenic and osteoclastogenic differentiation. Overall, IL-1β release in PDLSC pyroptosis both damaged the differentiation capability of circumjacent PDLSCs and promoted the formation of osteoclasts, which jointly aggravated the process of periodontitis.

A previous study revealed that intracellular LPS or inflammatory caspases were required for the activation of GSDMD (Rathinam et al., 2019). As elevated levels of LPS from gram-negative bacterial and inflammatory caspases were often detected in patients with periodontitis and in murine periodontitis, this provided a prerequisite for the activation of GSDMD and IL-1β release (Cheng et al., 2017; Rocha et al., 2020). In this work, we also confirmed that the activation of caspase-4/11 was elevated in human periodontitis samples and in murine periodontitis models and that *P. gingivalis* (a gram-negative periodontopathogen) induced PDLSC pyroptosis with the cleavage of GSDMD and IL-1β release. Overall, our data demonstrated that GSDMD-dominant PDLSC pyroptosis played a pivotal role in the pathological damage of periodontitis.

GSDMD has been reported to execute pyroptosis in inflammation and to control proinflammatory cytokine IL-1β release (Ding et al., 2016). The induction of GSDMD in human periodontitis and ligature-induced rat periodontitis and the restorative effect of IL-1β antibody and caspase-4 inhibitor in rat periodontitis led us to hypothesize that GSDMD-driven pyroptosis was probably the key determinant of periodontitis. Thus, we further addressed the functional significance of pyroptosis in periodontitis by knocking out *Gsdmd*, which is the vital executor of pyroptosis. Our data showed that knockout of *Gsdmd* expression significantly reduce the loss of alveolar bone and periodontal ligament damage. This reduction was associated with increased expression of the osteogenic genes and downregulated expression of osteoclastogenic genes. Collectively, these results indicate that GSDMD-driven pyroptosis is an indispensable mechanism involved in the pathogenesis of periodontitis.

In summary, the most important finding in this study was revealing a direct link between PDLSC pyroptosis and human/murine periodontitis. The initiation of PDLSC pyroptosis disrupts the homeostatic balance of bone formation and resorption by releasing IL-1β, consequently aggravating the occurrence of periodontitis (Figure 8). Our study provides a novel insight into the pathogenic mechanism of periodontitis, which could potentially be valuable to treatment.

## Data Availability Statement

The raw data supporting the conclusions of this article will be made available by the authors, without undue reservation.

## Ethics Statement

The studies involving human participants were reviewed and approved by the Research Ethics Committee of the Ninth People’s Hospital (approval NO. SH9H-2020-TK60-1). The patients/participants provided their written informed consent to participate in this study. The animal study was reviewed and approved by Animal Care and Use Committee of Shanghai Jiao Tong University Affiliated Ninth People’s Hospital (approval NO. SH9H-2020-A132-1). Written informed consent was obtained from the individual(s) for the publication of any potentially identifiable images or data included in this article.

## Author Contributions

QC, XGL, DZ, and CY: conception and design. JZ, LC, QX, XHL, SN, JLi, and GQ: provision of study material or patients. QC: collection and/or assembly of data. QC and DW: data analysis and interpretation. QC and XGL: manuscript writing. DZ and CY: final approval of manuscript. QC, JZ, JFL, and DZ: funding. All authors reviewed the results and approved the final version of the manuscript.

## Conflict of Interest

The authors declare that the research was conducted in the absence of any commercial or financial relationships that could be construed as a potential conflict of interest.
