# Supplementary material for: Periodontal Inflammation-Triggered by Periodontal Ligament Stem Cell Pyroptosis Exacerbates Periodontitis
Source: Front Cell Dev Biol. 2021 Apr 1;9:663037. doi: 10.3389/fcell.2021.663037 (PMC8049442; doi:10.3389/fcell.2021.663037)
Supplement: Supplementary file 1 [file Data_Sheet_1.PDF]

Supplementary Material

Supplementary Figure 1. Isolation and characterization of PDLSCs.

(A) PDLSCs were assessed and sorted by flow cytometry. PDLSCs were positive for CD44, CD90, CD73, and CD105. **(B)** Osteogenic differentiation of PDLSCs and staining with Alizarin red. Alizarin red staining demonstrated mineral deposits after 21 days of osteogenic induction. **(C)** Adipogenic differentiation of PDLSCs and staining with Oil red. Oil red staining revealed oil droplet-filled adipocyte-like cells after 21 days of adipogenic induction. **(D)** Chondrogenic differentiation for 30 days and staining with Alcian Blue. Representative data showing Alcian Blue-stained pellets after histological processing. **(E)** Expression of the osteogenic markers RUNX2, ALP, and OPN in PDLSCs was detected after osteogenic induction by western blot. **(F)** Expression of adipogenic markers APN, PPAR-γ, and C/EBP-α in PDLSCs was detected after adipogenic differentiation by western blot. GAPDH was used as the loading control. Control, PDLSC basic culture media without differentiation induction.

Supplementary Figure 2. The paracrine effect of PDLSC pyroptosis on adipogenic differentiation.

(A) Schematic diagram of coculture design indicating the placement of PDLSCs with LPS treatment, with LPS^{FuGene} treatment, or without treatment on a transwell insert and lower chamber consisting of healthy PDLSCs. **(B-H)** Cells in the cocultured transwell system were maintained in adipogenic differentiation medium for 21 days. PDLSCs in a single culture (without coculture) were maintained in MEM-α medium as a control.

(B) Representative images of Oil red O staining after culturing in adipogenic differentiation medium. **(C)** Quantification of the Oil red O staining results. **(D)** Immunoblots for APN, PPAR-γ, and C/EBP-α in whole-cell lysates. GAPDH was used as a loading control. *APN* **(E)**, *PPAR-γ* **(F)**, *C/EBP-α* **(G)**, and *FABP4* **(H)** mRNAs were subjected to real-time PCR analysis, and the expression levels were normalized to that of *36B4*. The data are presented as the mean ± SEM. P values were determined by the unpaired two-tailed t-test with Welch's correction. *P< 0.05, **P< 0.01.

Supplementary Figure 3. IL-1β was the key factor in the regulatory role of PDLSC pyroptosis in adipogenic differentiation.

(A-F) PDLSCs were incubated with adipogenic differentiation medium for 21 days with or without 500 pg/ml IL-1β treatment. **(A)** Representative images of Oil red staining. **(B)** Quantification of the oil red staining results. *APN* **(C)**, *PPAR-γ* **(D)**, *C/EBP-α* **(E)**, and *FABP4* **(F)** mRNAs were subjected to real-time PCR analysis, and the expression levels were normalized to that of *36B4*. The data are presented as the mean ± SEM. P values were determined by the unpaired two-tailed t-test with Welch's correction. *P< 0.05, **P< 0.01.

Supplementary Figure 4. The rescue effect of IL-1β antibody on the pyroptotic PDLSC-induced inflammatory microenvironment.

(A) hPDLSCs cocultured with LPS-treated PDLSCs, LPS^{FuGene}-treated PDLSCs, or LPS^{FuGene}-treated PDLSCs plus IL-1β antibody incubation were maintained in

osteogenic differentiation medium. Micrographs of alizarin red staining after culturing in osteogenic differentiation medium for 21 days. **(B)** THP-1 cells cocultured with LPS-treated PDLSCs, LPS^{FuGene}-treated PDLSCs, or LPS^{FuGene}-treated PDLSCs plus IL-1β antibody incubation were maintained in osteoclast differentiation medium. Micrographs of TRAP staining after culturing in osteoclast differentiation medium for 14 days.

Supplementary Figure 5. Construction of Gsdmd-/- mice.

(A) Silk thread was used to form a ligature around the left maxillary first molar for 2 weeks to induce periodontitis. **(B)** Schematic diagram of the Gsdmd gene knockout strategy in mice. **(C)** Genotyping identified the Gsdmd gene knockout efficiency achieved by CRISPR/Cas-9 technology. **(D)** The expression levels of Gsdmd in periodontal tissues of Gsdmd-/- and WT mice were determined by western blotting.

Supplementary Table 1: Primary antibody list.

Supplementary Table 2: Sequences of qPCR primers.

Figure S1

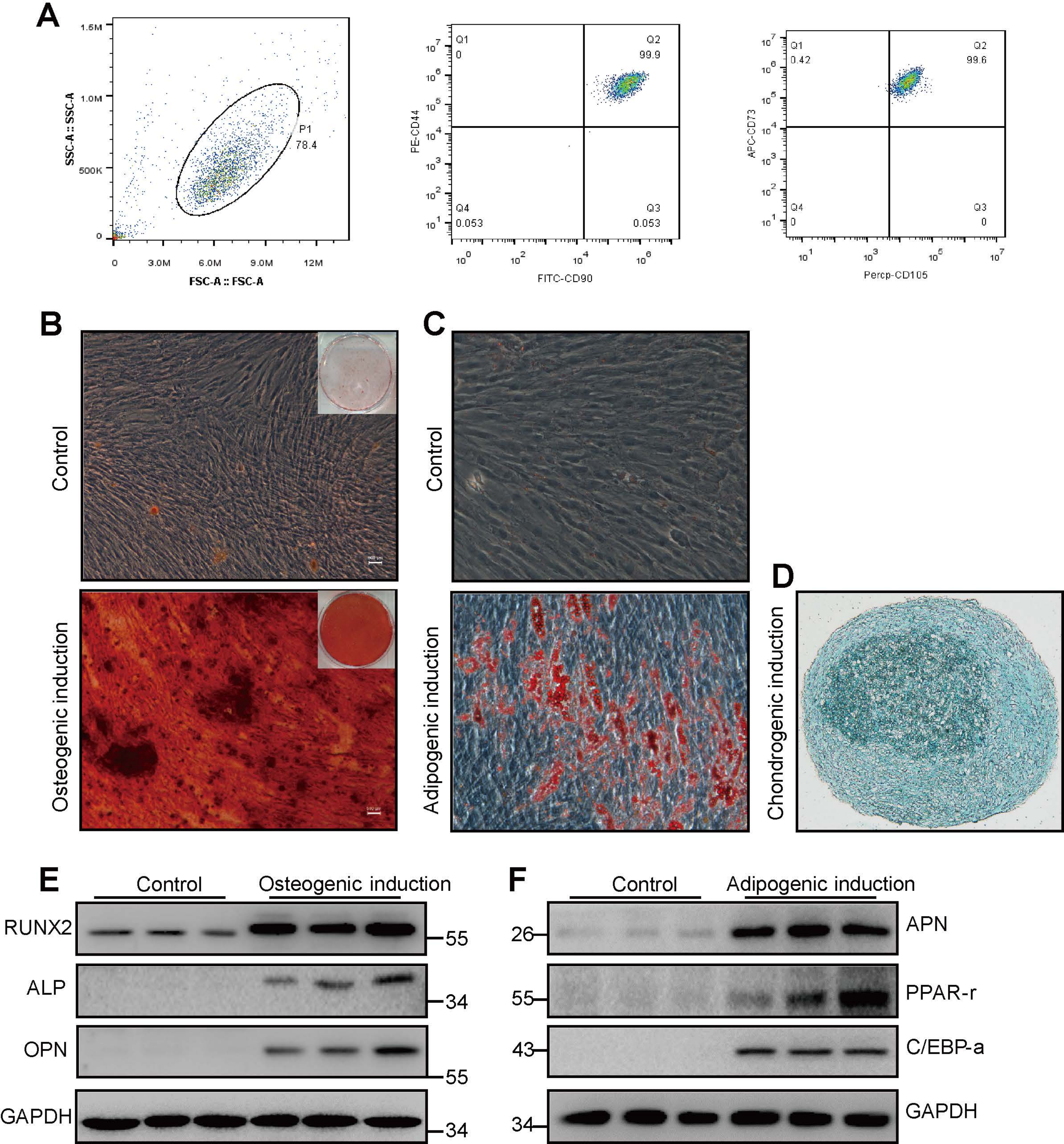

A
SSC-A :: SSC-A
FSC-A :: FSC-A
P1
78.4
PE-CD44
FITC-CD90
Q1 0
Q2 99.9
Q4 0.053
Q3 0.053
APC-CD73
Percp-CD105
Q1 0.42
Q2 99.6
Q4 0
Q3 0
B
Control
Osteogenic induction
C
Control
Adipogenic induction
D
Chondrogenic induction
E
Control
Osteogenic induction
RUNX2
ALP
OPN
GAPDH
55
34
55
34
F
Control
Adipogenic induction
APN
PPAR-r
C/EBP-a
GAPDH
26
55
43
34

Figure S2

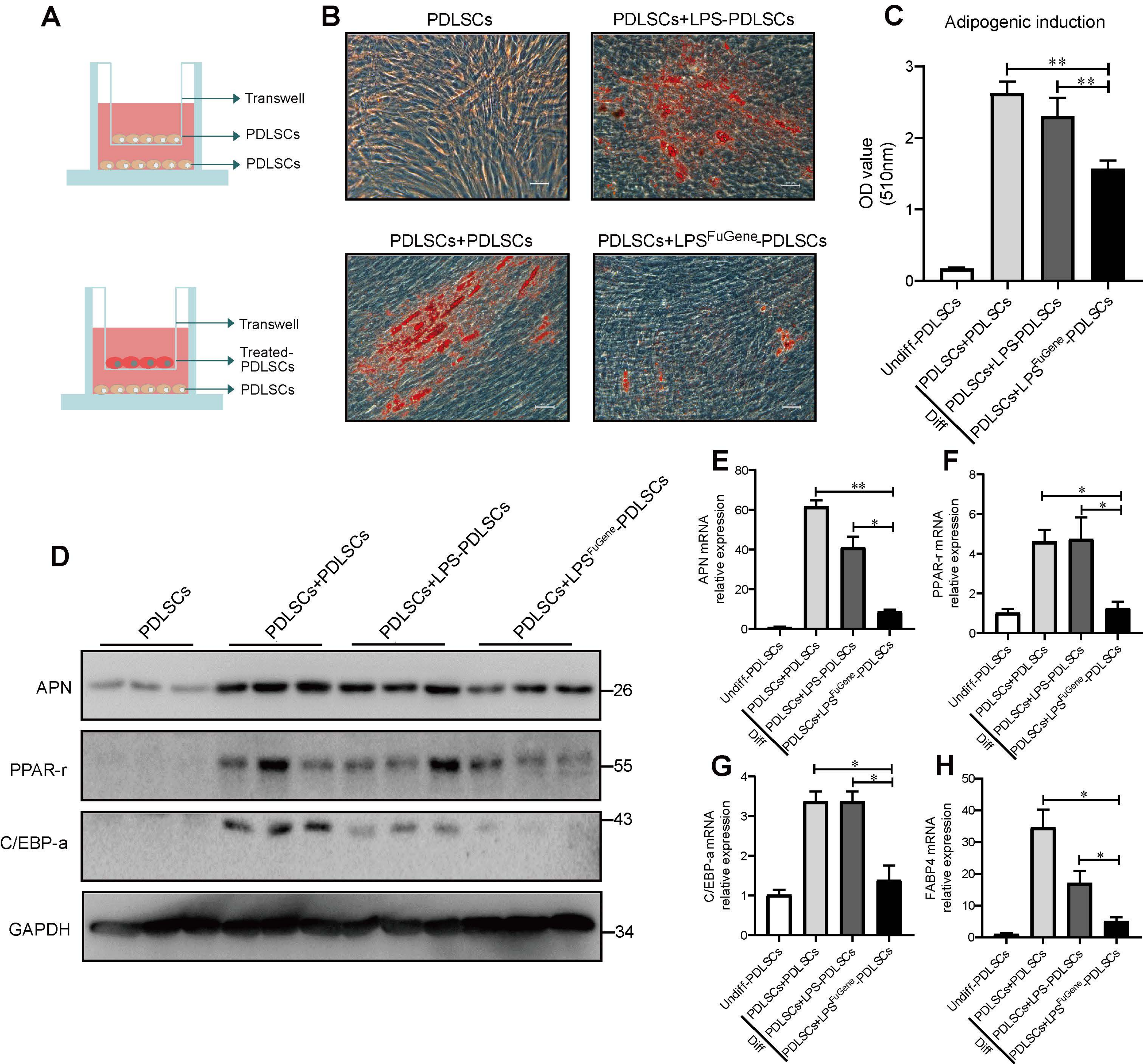
A
Transwell
PDLSCs
PDLSCs
Transwell
Treated-PDLSCs
PDLSCs
B
PDLSCs
PDLSCs+LPS-PDLSCs
PDLSCs+PDLSCs
PDLSCs+LPS^FuGene-PDLSCs
C
Adipogenic induction
OD value (510nm)
Undiff-PDLSCs
PDLSCs+PDLSCs
PDLSCs+LPS-PDLSCs
PDLSCs+LPS^FuGene-PDLSCs
Diff
D
PDLSCs
PDLSCs+PDLSCs
PDLSCs+LPS-PDLSCs
PDLSCs+LPS^FuGene-PDLSCs
APN
PPAR-r
C/EBP-a
GAPDH
26
55
43
34
E
APN mRNA relative expression
F
PPAR-r mRNA relative expression
G
C/EBP-a mRNA relative expression
H
FABP4 mRNA relative expression

Figure S3

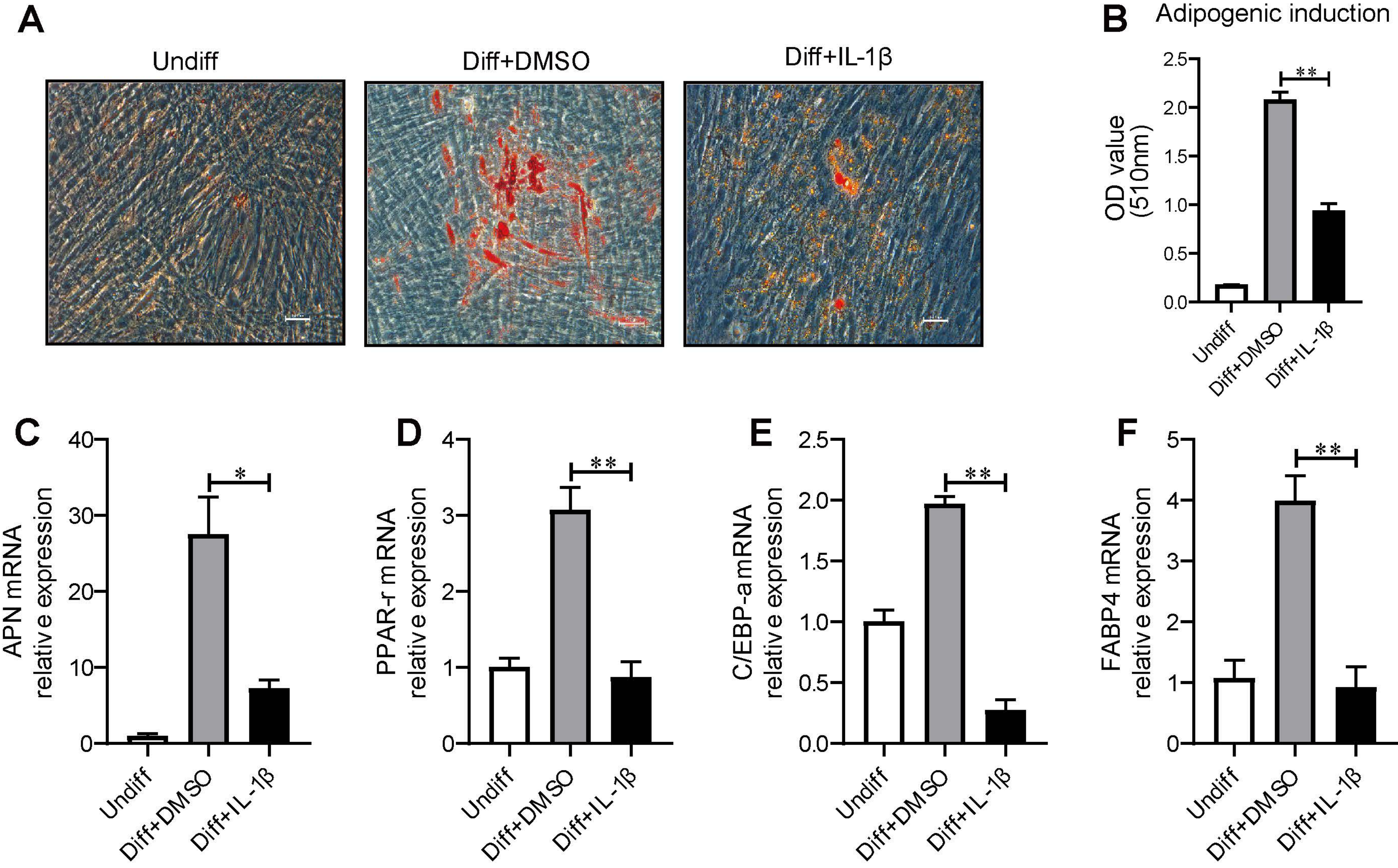

A
Undiff
Diff+DMSO
Diff+IL-1β
B
Adipogenic induction
OD value (510nm)
2.5
2.0
1.5
1.0
0.5
0.0
**
Undiff
Diff+DMSO
Diff+IL-1β
C
APN mRNA relative expression
40
30
20
10
0
*
D
PPAR-r mRNA relative expression
4
3
2
1
0
**
E
C/EBP-amRNA relative expression
2.5
2.0
1.5
1.0
0.5
0.0
**
F
FABP4 mRNA relative expression
5
4
3
2
1
0
**
Undiff
Diff+DMSO
Diff+IL-1β

Figure S4

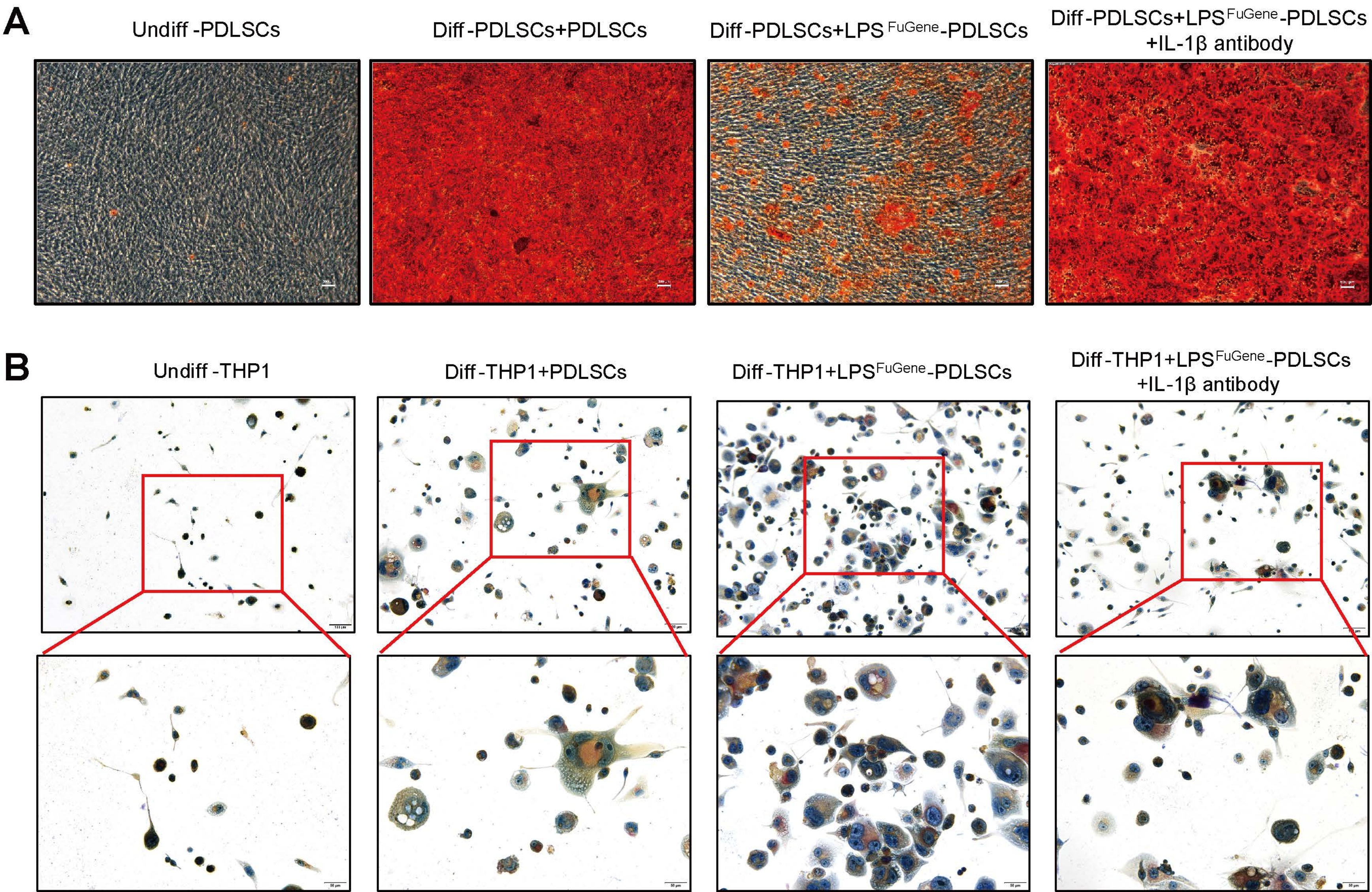
A
Undiff -PDLSCs
Diff -PDLSCs+PDLSCs
Diff -PDLSCs+LPS FuGene-PDLSCs
Diff -PDLSCs+LPS FuGene-PDLSCs +IL-1β antibody
B
Undiff -THP1
Diff -THP1+PDLSCs
Diff -THP1+LPS FuGene-PDLSCs
Diff -THP1+LPS FuGene-PDLSCs +IL-1β antibody

Figure S5

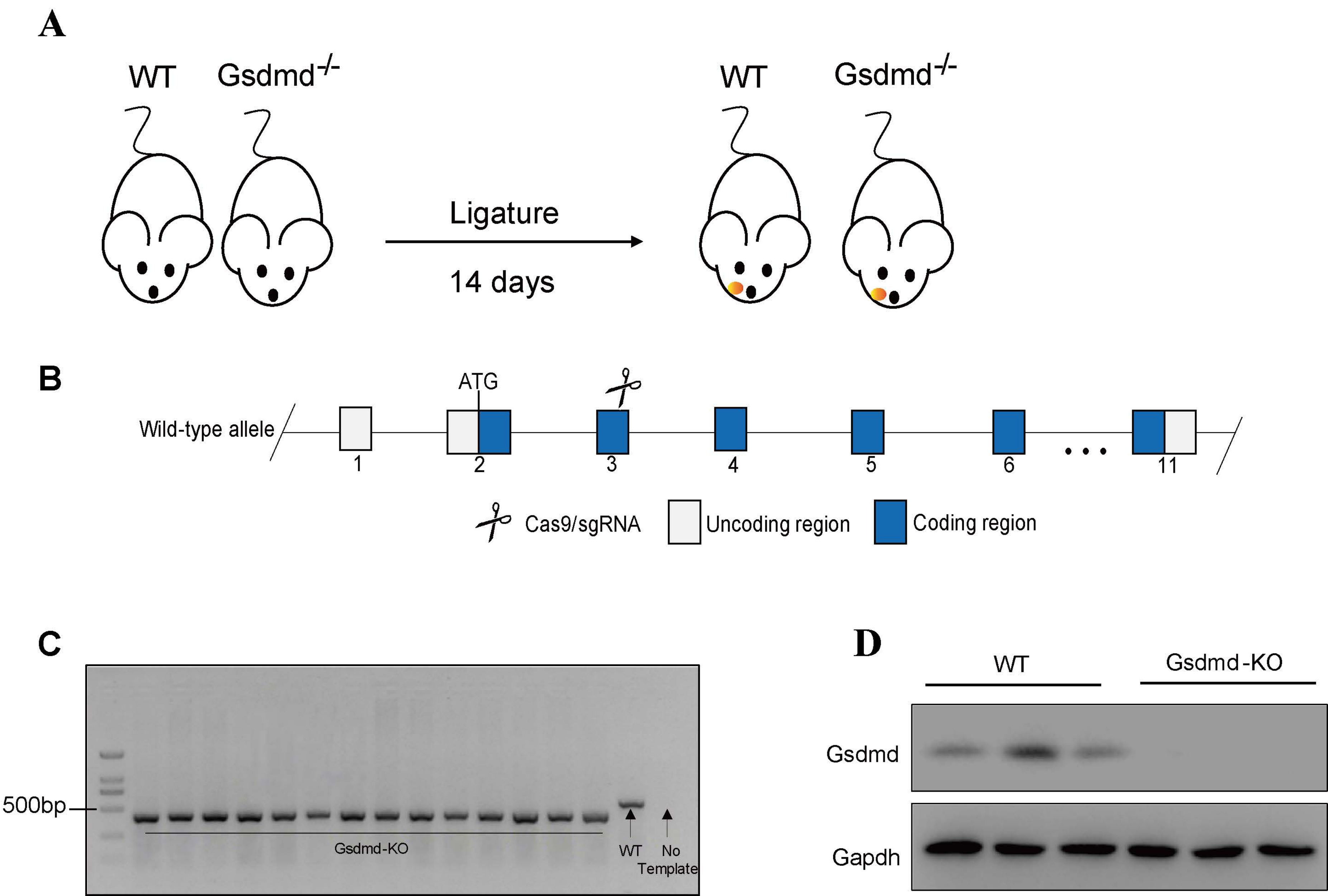
A
WT
Gsdmd-/-
Ligature
14 days
WT
Gsdmd-/-
B
ATG
Wild-type allele
1
2
3
4
5
6
11
Cas9/sgRNA
Uncoding region
Coding region
C
500bp
Gsdmd-KO
WT
No Template
D
WT
Gsdmd-KO
Gsdmd
Gapdh

Table S1: Primary antibody list

Target	Catalog No.	Company
GSDMD	G7422	Sigma-Aldrich
IL-1β	AF-401-NA	R&D Systems
CD90	Ab133350	Abcam
CD90	Ab181469	Abcam
Caspase-4	M029-3	MBL
Caspase-1	#4199	Cell signaling technology
NLRP3	#13158	Cell signaling technology
RUNX2	Ab23981	Abcam
ALP	Ab83259	Abcam
OPN	Ab8448	Abcam
APN (Adiponectin)	#2789	Cell signaling technology
PPAR-γ	#2435	Cell signaling technology
C/EBP-α	#8178	Cell signaling technology
GAPDH	Ab181602	Abcam

Table S2: Sequences of qPCR primers

Primer nam(	Forward (5'-3')	Reverse (5'-3')
Human-specific primers		
RUNX2	5’-TGGTTACTGTCATGGCGGGTA -3’	5’- TCTCAGATCGTTGAACCTTGCTA-3’
ALP	5’-GTGAACCGCAACTGGTACTC-3’	5’-GAGCTGCGTAGCGATGTCC-3’
OPN	5’-GAAGTTTCGCAGACCTGACAT-3’	5’-GTATGCACCATTCAACTCCTCG-3’
OSX	5’-CCTCTGCGGGACTCAACAAC-3’	5’-AGCCCATTAGTGCTTGTAAAGG-3’
TRAP	5’-GACTGTGCAGATCCTGGGTG-3’	5’-GGTCAGAGAATACGTCCTCAAAG-3’
CTSK	5’-ACTCAAAGTACCCCTGTCTCAT-3	5’-CCACAGAGCTAAAAGCCCAAC-3’
DCSTAMP	5’-CCTTGCCACTCCACTAAGTGT-3’	5’-CTCTGTGGTTGTTGCCATCTG-3’
NFATC1	5’-CACCGCATCACAGGGAAGAC-3’	5’-GCACAGTCAATGACGGCTC-3’
APN	5’-GGCTTTCCGGGAATCCAAGG-3’	5’-TGGGGATAGTAACGTAAGTCTCC-3’
PPAR-γ	5’-GTCGTGCAGGAGATCACAGA-3’	5’-GGGCTCCATAAAGTCACCAA-3’
C/EBP-α	5’-GTGGACAAGAACAGCAACGA -3’	5’-GGTCATTGTCACTGGTCAGC-3’
FABP4	5’-ACTGGGCCAGGAATTTGACG-3’	5’-CTCGTGGAAGTGACGCCTT-3’
IL-1β	5’-ATGATGGCTTATTACAGTGGCAA	5’-GTCGGAGATTCGTAGCTGGA-3’
IL-2	5’-AACTCCTGTCTTGCATTGCAC-3’	5’-GCTCCAGTTGTAGCTGTGTTT-3’
IL-4	5’-CCAACTGCTTCCCCCTCTG-3’	5’-TCTGTTACGGTCAACTCGGTG-3’
IL-6	5’-ACTCACCTCTTCAGAACGAATTG-	5’-CCATCTTTGGAAGGTTCAGGTTG-3’
IL-8	5’- ACTGAGAGTGATTGAGAGTGGA(	5’- AACCCTCTGCACCCAGTTTTC-3’
IL-10	5’- GACTTTAAGGGTTACCTGGGTTG	5’- TCACATGCGCCTTGATGTCTG-3’
IL-12	5’-ACCCTGACCATCCAAGTCAAA-3’	5’-TTGGCCTCGCATCTTAGAAAG-3’
IL-17α	5’-TCCCACGAAATCCAGGATGC-3’	5’-GGATGTTCAGGTTGACCATCAC-3’
IL-18	5’-TCTTCATTGACCAAGGAAATCGG	5’-TCCGGGGTGCATTATCTCTAC-3’
IL-22	5’- GCTTGACAAGTCCAACTTCCA-3’	5’- GCTCACTCATACTGACTCCGT-3’
IFN-α	5’-ATCTCAGCAAGCCCAGAAGT-3’	5’-GGGTCTCAGGGAGATCACAG-3’
IFN-γ	5’-TCGGTAACTGACTTGAATGTCCA-	5’-TCGCTTCCCTGTTTTAGCTGC-3’
TNF-α	5’-CCTCTCTCTAATCAGCCCTCTG-3’	5’-GAGGACCTGGGAGTAGATGAG-3’
36B4	5’-AGCCCAGAACACTGGTCTC-3’	5’-ACTCAGGATTTCAATGGTGCC-3’
Mouse-specific primers		
ALP	5’-CCAACTCTTTTGTGCCAGAGA-3’	5’-GGCTACATTGGTGTTGAGCTTTT-3’
Osx	5’-ATGGCGTCCTCTCTGCTTG-3’	5’-TGAAAGGTCAGCGTATGGCTT-3’
Runx2	5’-ATGCTTCATTCGCCTCACAAA-3’	5’-GCACTCACTGACTCGGTTGG-3’
Opn	5’-AGCAAGAAACTCTTCCAAGCAA-:	5’-GTGAGATTCGTCAGATTCATCCG-3’
Nfatc1	5’-GACCCGGAGTTCGACTTCG-3’	5’-TGACACTAGGGGACACATAACTG-3’
Dcstamp	5’-GGGGACTTATGTGTTTCCACG-3’	5’-ACAAAGCAACAGACTCCCAAAT-3’
Acp5(Trap)	5’-CACTCCCACCCTGAGATTTGT-3’	5’-CATCGTCTGCACGGTTCTG-3’
Ctsk	5’-GAAGAAGACTCACCAGAAGCAG-	5’-TCCAGGTTATGGGCAGAGATT-3’
Traf5	5’-TTTGAGCCCGACACCGAGTA-3’	5’-AGAGACCGGATGCACTGCT-3’
36b4	5’-AGATTCGGGATATGCTGTTGGC-3	5’-TCGGGTCCTAGACCAGTGTTC-3’
